# Supplementary figures and images for: Investigation for a multi-silique trait in Brassica napus by alternative splicing analysis
Source: PeerJ. 2020 Oct 8;8:e10135. doi: 10.7717/peerj.10135 (PMC7548069; doi:10.7717/peerj.10135)

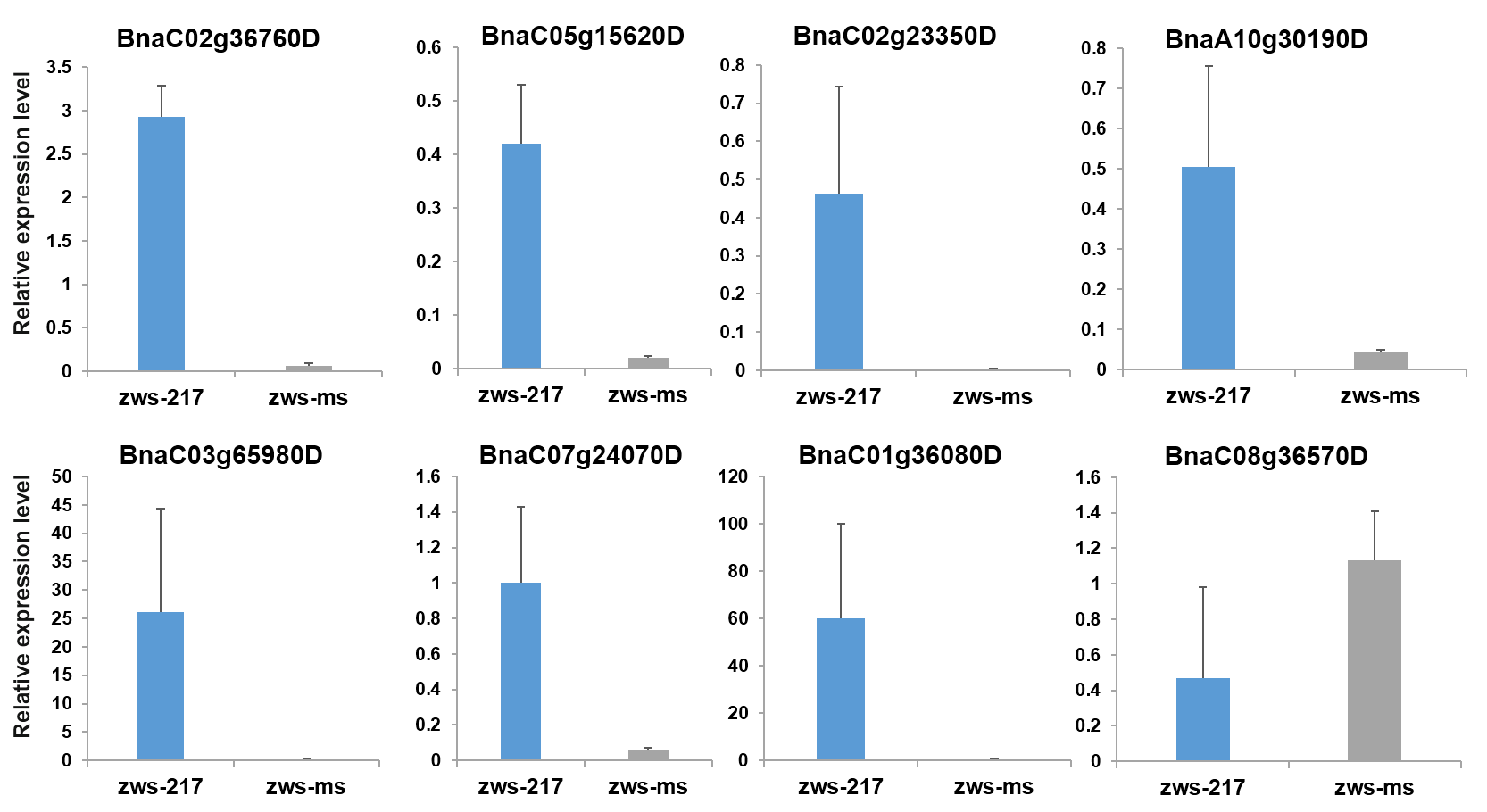

Supplement: Supplemental Information 1 — BnACTIN used as the internal control. The primers for the qPCR were BnACTIN: 5’-CTGGAATTGCTGACCGTATGAG-3’ and 5’-GCCAAGATGGATCCTCCAATC-3’; BnaC08g36570D: 5’-TTACTATCAAGAAGACGACTCAA-3’ and 5’-GCCTCTCCACAACCAAAT-3’; BnaC02g36760D: 5’-TTAATGGCGGTTAAGTCAA-3’ and 5’-CGAAGTCAACGGATTCTT-3’; BnaC05g15620D: 5’-AAAGGAGCAGCCATTGTG-3’ and 5’-GCAGAATCAGCCATCATAGT-3’; BnaC02g23350D: 5’-CAGGATTTAGTAATGCGAACAA-3’ and 5’-CCGAGGGATGAACAAAGT-3’; BnaA10g30190D: 5’-TATTGGCATTCGCTGGTA-3’ and 5’-GAGAATCGGACTGAATAGGA-3’; BnaC03g65980D: 5’-CGGTTCGGTTCTCTACATC-3’ and 5’-ATGCGTTGGTGGAAGGTA-3’; BnaC07g24070D: 5’-ACTTTAGCAGCACAGCAT-3’ and 5’-TTGATCGCCGACTTTGAA-3’; BnaC01g36080D: 5’-TTCACATTACTCTTCCTCTTCTT-3’ and 5’-AACACGCAAGCAATCAAC-3’. [file peerj-08-10135-s001.png]
