# Supplementary material for: Investigation for a multi-silique trait in Brassica napus by alternative splicing analysis
Source: PeerJ. 2020 Oct 8;8:e10135. doi: 10.7717/peerj.10135 (PMC7548069; doi:10.7717/peerj.10135)
Supplement: Supplemental Information 2 — Note: T01, T02, and T03: Buds from three independent zws-ms plants at the budding stage; T04, T05, and T06: Buds of three independent zws-217 plants at the budding stage. [file peerj-08-10135-s002.docx]

Table S1. Summary of the transcriptome sequencing data from Xindu

| Line | Sample ID | Number of clean reads | Number of clean bases | GC content (%) | % ≥ Q30 |
| --- | --- | --- | --- | --- | --- |
| zws-ms | T01 | 27,988,798 | 8,342,636,670 | 46.67 | 89.85% |
|  | T02 | 33,972,932 | 10,138,074,420 | 47.72 | 91.58% |
|  | T03 | 41,826,443 | 12,487,251,074 | 47.51 | 91.62% |
| zws-217 | T04 | 39,062,063 | 11,657,288,984 | 47.18 | 89.73% |
|  | T05 | 41,486,437 | 12,383,790,994 | 47.21 | 90.36% |
|  | T06 | 35,568,484 | 10,595,114,562 | 47.08 | 90.12% |

Note: T01, T02, and T03: Buds from three independent zws-ms plants at the budding stage; T04, T05, and T06: Buds of three independent zws-217 plants at the budding stage.
