# Supplementary material for: Investigation for a multi-silique trait in Brassica napus by alternative splicing analysis
Source: PeerJ. 2020 Oct 8;8:e10135. doi: 10.7717/peerj.10135 (PMC7548069; doi:10.7717/peerj.10135)
Supplement: Supplemental Information 4 — Note: T01, T02, and T03: Buds from three independent zws-ms plants at the budding stage; T04, T05, and T06: Buds of three independent zws-217 plants at the budding stage. [file peerj-08-10135-s004.docx]

Table S3. Summary of the transcriptome sequencing data from cold environment Ma’erkang

| Line | Sample ID | Number of clean reads | Number of clean bases | GC content (%) | % ≥ Q30 |
| --- | --- | --- | --- | --- | --- |
| zws-ms | T01 | 26,966,557 | 8,065,592,854 | 46.10 | 92.69 |
|  | T02 | 20,951,861 | 6,265,313,130 | 46.25 | 92.91 |
|  | T03 | 21,220,925 | 6,346,221,680 | 46.22 | 92.77 |
| zws-217 | T04 | 21,198,442 | 6,338,330,810 | 46.26 | 93.34 |
|  | T05 | 22,846,386 | 6,831,542,002 | 46.52 | 93.16 |
|  | T06 | 24,326,347 | 7,273,053,016 | 46.24 | 92.81 |

Note: T01, T02, and T03: Buds from three independent zws-ms plants at the budding stage; T04, T05, and T06: Buds of three independent zws-217 plants at the budding stage.
