# Supplementary material for: Investigation for a multi-silique trait in Brassica napus by alternative splicing analysis
Source: PeerJ. 2020 Oct 8;8:e10135. doi: 10.7717/peerj.10135 (PMC7548069; doi:10.7717/peerj.10135)
Supplement: Supplemental Information 5 — Note: T01, T02, and T03: Buds from three independent zws-ms plants at the budding stage; T04, T05, and T06: Buds of three independent zws-217 plants at the budding stage. [file peerj-08-10135-s005.docx]

Table S4. Mapped reads from the transcriptome sequencing data from cold environment Ma’erkang.

| Sample | Total reads | Mapped reads | Unique mapped reads | Multiple mapped reads | Reads mapped to '+' | Reads mapped to '-' |
| --- | --- | --- | --- | --- | --- | --- |
| T01 | 53,933,114 | 47,831,347 (88.69%) | 45,441,502 (84.26%) | 2,389,845 (4.43%) | 23,518,714 (43.61%) | 23,652,130 (43.85%) |
| T02 | 41,903,722 | 37,821,153 (90.26%) | 35,924,318 (85.73%) | 1,896,835 (4.53%) | 18,599,926 (44.39%) | 18,705,082 (44.64%) |
| T03 | 42,441,850 | 37,620,515 (88.64%) | 35,785,300 (84.32%) | 1,835,215 (4.32%) | 18,497,596 (43.58%) | 18,600,530 (43.83%) |
| T04 | 42,396,884 | 37,761,798 (89.07%) | 35,925,759 (84.74%) | 1,836,039 (4.33%) | 18,554,209 (43.76%) | 18,660,596 (44.01%) |
| T05 | 45,692,772 | 40,224,798 (88.03%) | 38,220,526 (83.65%) | 2,004,272 (4.39%) | 19,747,558 (43.22%) | 19,871,445 (43.49%) |
| T06 | 48,652,694 | 43,146,823 (88.68%) | 41,165,652 (84.61%) | 1,981,171 (4.07%) | 21,222,437 (43.62%) | 21,337,467 (43.86%) |

Note: T01, T02, and T03: Buds from three independent zws-ms plants at the budding stage; T04, T05, and T06: Buds of three independent zws-217 plants at the budding stage.
