# Supplementary material for: Investigation for a multi-silique trait in Brassica napus by alternative splicing analysis
Source: PeerJ. 2020 Oct 8;8:e10135. doi: 10.7717/peerj.10135 (PMC7548069; doi:10.7717/peerj.10135)
Supplement: Supplemental Information 7 [file peerj-08-10135-s007.docx]

Table S6. The expression level and annotation of AS genes from plants grown in Ma’erkang.

| Gene ID | log_2_FC | FDR for FC | regulated | GO annotation | KEGG pathway |
| --- | --- | --- | --- | --- | --- |
| BnaA01g22780D | -4.564305 | 9.75E-26 | down | MF: transcription coactivator activity (GO:0003713); CC: cytoplasm (GO:0005737); MF: zinc ion binding (GO:0008270); | -- |
| BnaA01g26600D | -2.659898 | 8.20E-14 | down | CC: nucleus (GO:0005634); CC: Golgi apparatus (GO:0005794); BP: starch metabolic process (GO:0005982); BP: plant-type secondary cell wall biogenesis (GO:0009834); BP: glucuronoxylan biosynthetic process (GO:0010417); MF: glucuronoxylan glucuronosyltransferase activity (GO:0080116); | -- |
| BnaA01g28890D | -3.243022 | 1.14E-12 | down | BP: protein glycosylation (GO:0006486); MF: Ran GTPase binding (GO:0008536); | -- |
| BnaA02g02700D | -2.411674 | 5.34E-06 | down | CC: vacuolar membrane (GO:0005774); CC: plasma membrane (GO:0005886); BP: plant-type secondary cell wall biogenesis (GO:0009834); BP: xylem development (GO:0010089); BP: cellulose microfibril organization (GO:0010215); BP: glucuronoxylan metabolic process (GO:0010413); BP: cell growth (GO:0016049); CC: anchored component of membrane (GO:0031225); BP: xylan biosynthetic process (GO:0045492); | -- |
| BnaA02g06580D | -2.169703 | 2.34E-06 | down | MF: copper ion binding (GO:0005507); BP: lignin biosynthetic process (GO:0009809); BP: plant-type cell wall biogenesis (GO:0009832); BP: glucuronoxylan metabolic process (GO:0010413); BP: xylan biosynthetic process (GO:0045492); BP: lignin catabolic process (GO:0046274); CC: apoplast (GO:0048046); MF: hydroquinone:oxygen oxidoreductase activity (GO:0052716); BP: oxidation-reduction process (GO:0055114); | -- |
| BnaA02g06740D | -2.151418 | 3.21E-05 | down | CC: extracellular region (GO:0005576); BP: response to gibberellin (GO:0009739); | -- |
| BnaA02g11730D | -2.807638 | 2.25E-08 | down | MF: copper ion binding (GO:0005507); CC: extracellular region (GO:0005576); CC: plant-type cell wall (GO:0009505); CC: plasmodesma (GO:0009506); MF: oxidoreductase activity (GO:0016491); BP: oxidation-reduction process (GO:0055114); | -- |
| BnaA02g14070D | -2.863193 | 5.39E-08 | down | CC: extracellular region (GO:0005576); BP: pollen exine formation (GO:0010584); | -- |
| BnaA03g00780D | -2.042957 | 1.94E-05 | down | CC: plasma membrane (GO:0005886); BP: plant-type secondary cell wall biogenesis (GO:0009834); CC: anchored component of membrane (GO:0031225); | -- |
| BnaA03g52460D | -3.357548 | 1.15E-15 | down | CC: extracellular region (GO:0005576); BP: lipid transport (GO:0006869); MF: lipid binding (GO:0008289); | -- |
| BnaA03g58390D | -2.251668 | 2.97E-09 | down | CC: cell wall (GO:0005618); CC: plasma membrane (GO:0005886); BP: response to osmotic stress (GO:0006970); BP: response to water deprivation (GO:0009414); BP: plant-type secondary cell wall biogenesis (GO:0009834); BP: salicylic acid mediated signaling pathway (GO:0009863); BP: jasmonic acid mediated signaling pathway (GO:0009867); BP: ethylene-activated signaling pathway (GO:0009873); BP: positive regulation of abscisic acid biosynthetic process (GO:0010116); BP: glucuronoxylan metabolic process (GO:0010413); CC: integral component of membrane (GO:0016021); MF: cellulose synthase (UDP-forming) activity (GO:0016760); BP: cellulose biosynthetic process (GO:0030244); BP: defense response to bacterium (GO:0042742); BP: xylan biosynthetic process (GO:0045492); BP: defense response to fungus (GO:0050832); BP: cell wall thickening (GO:0052386); | -- |
| BnaA05g06610D | -2.425077 | 2.11E-08 | down | MF: copper ion binding (GO:0005507); BP: lignin biosynthetic process (GO:0009809); BP: plant-type secondary cell wall biogenesis (GO:0009834); BP: glucuronoxylan metabolic process (GO:0010413); BP: xylan biosynthetic process (GO:0045492); BP: lignin catabolic process (GO:0046274); CC: apoplast (GO:0048046); MF: hydroquinone:oxygen oxidoreductase activity (GO:0052716); BP: oxidation-reduction process (GO:0055114); | -- |
| BnaA05g07340D | -2.73252 | 7.84E-10 | down | CC: Golgi apparatus (GO:0005794); BP: plant-type secondary cell wall biogenesis (GO:0009834); BP: glucuronoxylan biosynthetic process (GO:0010417); MF: galactosylgalactosylxylosylprotein 3-beta-glucuronosyltransferase activity (GO:0015018); CC: membrane (GO:0016020); MF: xylosyltransferase activity (GO:0042285); | -- |
| BnaA05g24130D | -2.042065 | 2.51E-15 | down | -- | -- |
| BnaA05g28590D | -2.024571 | 2.10E-06 | down | CC: nucleus (GO:0005634); BP: response to water deprivation (GO:0009414); BP: response to salt stress (GO:0009651); BP: positive regulation of transcription, DNA-templated (GO:0045893); BP: response to freezing (GO:0050826); | -- |
| BnaA06g14250D | -3.544127 | 2.84E-20 | down | CC: extracellular region (GO:0005576); BP: lipid metabolic process (GO:0006629); MF: lipase activity (GO:0016298); | -- |
| BnaA06g31090D | -2.075301 | 2.68E-05 | down | -- | -- |
| BnaA06g32330D | -3.181766 | 1.81E-25 | down | MF: copper ion binding (GO:0005507); CC: plasma membrane (GO:0005886); MF: electron carrier activity (GO:0009055); BP: glucuronoxylan metabolic process (GO:0010413); CC: anchored component of membrane (GO:0031225); BP: xylan biosynthetic process (GO:0045492); | -- |
| BnaA06g36370D | -2.101645 | 4.19E-09 | down | CC: plasma membrane (GO:0005886); MF: zinc ion binding (GO:0008270); BP: plant-type secondary cell wall biogenesis (GO:0009834); BP: salicylic acid mediated signaling pathway (GO:0009863); BP: jasmonic acid mediated signaling pathway (GO:0009867); BP: ethylene-activated signaling pathway (GO:0009873); BP: glucuronoxylan metabolic process (GO:0010413); CC: integral component of membrane (GO:0016021); MF: cellulose synthase (UDP-forming) activity (GO:0016760); BP: cellulose biosynthetic process (GO:0030244); BP: defense response to bacterium (GO:0042742); BP: xylan biosynthetic process (GO:0045492); BP: defense response to fungus (GO:0050832); BP: cell wall thickening (GO:0052386); | -- |
| BnaA06g39760D | -2.390628 | 7.64E-06 | down | CC: chloroplast (GO:0009507); BP: embryo development ending in seed dormancy (GO:0009793); BP: pollen exine formation (GO:0010584); | -- |
| BnaA07g04500D | 2.9096543 | 7.45E-14 | up | CC: mitochondrion (GO:0005739); | -- |
| BnaA07g19340D | -2.942241 | 3.40E-10 | down | CC: endoplasmic reticulum lumen (GO:0005788); CC: plasma membrane (GO:0005886); BP: protein folding (GO:0006457); BP: ER-nucleus signaling pathway (GO:0006984); BP: response to heat (GO:0009408); BP: systemic acquired resistance (GO:0009627); BP: response to high light intensity (GO:0009644); MF: heat shock protein binding (GO:0031072); BP: response to endoplasmic reticulum stress (GO:0034976); BP: response to hydrogen peroxide (GO:0042542); MF: unfolded protein binding (GO:0051082); BP: pathogen-associated molecular pattern dependent induction by symbiont of host innate immune response (GO:0052033); | Protein processing in endoplasmic reticulum (ko04141) |
| BnaA07g22160D | -2.296443 | 2.40E-05 | down | MF: iron ion binding (GO:0005506); CC: mitochondrion (GO:0005739); BP: spermidine metabolic process (GO:0008216); MF: electron carrier activity (GO:0009055); BP: pollen exine formation (GO:0010584); MF: oxygen binding (GO:0019825); MF: heme binding (GO:0020037); BP: oxidation-reduction process (GO:0055114); MF: tricoumaroylspermidine meta-hydroxylase activity (GO:0072547); MF: dicoumaroyl monocaffeoyl spermidine meta-hydroxylase activity (GO:0072548); MF: monocoumaroyl dicaffeoyl spermidine meta-hydroxylase activity (GO:0072549); MF: triferuloylspermidine meta-hydroxylase activity (GO:0072550); MF: diferuloyl mono-(hydroxyferuloyl) spermidine meta-hydroxylase activity (GO:0072551); MF: monoferuloyl di-(hydroxyferuloyl) spermidine meta-hydroxylase activity (GO:0072552); | Phenylpropanoid biosynthesis (ko00940) |
| BnaA07g27370D | -2.57877 | 2.65E-08 | down | CC: plasma membrane (GO:0005886); CC: anchored component of membrane (GO:0031225); | -- |
| BnaA07g37340D | 2.3380332 | 1.90E-09 | up | -- | -- |
| BnaA08g07600D | -2.52153 | 2.33E-06 | down | MF: hydrolase activity, hydrolyzing O-glycosyl compounds (GO:0004553); CC: plasma membrane (GO:0005886); BP: carbohydrate metabolic process (GO:0005975); BP: embryo development ending in seed dormancy (GO:0009793); BP: pollen exine formation (GO:0010584); MF: cation binding (GO:0043169); | -- |
| BnaA08g11810D | -3.395747 | 1.38E-13 | down | CC: extracellular region (GO:0005576); BP: lipid transport (GO:0006869); MF: lipid binding (GO:0008289); | -- |
| BnaA08g18970D | -2.010681 | 0.000322157 | down | CC: Golgi apparatus (GO:0005794); BP: plant-type secondary cell wall biogenesis (GO:0009834); BP: glucuronoxylan biosynthetic process (GO:0010417); CC: membrane (GO:0016020); MF: glucuronoxylan glucuronosyltransferase activity (GO:0080116); | -- |
| BnaA09g06740D | 4.6624665 | 2.47E-28 | up | CC: nucleus (GO:0005634); | -- |
| BnaA09g23510D | -2.462999 | 2.65E-10 | down | CC: extracellular region (GO:0005576); CC: mitochondrion (GO:0005739); CC: Golgi apparatus (GO:0005794); BP: lignin metabolic process (GO:0009808); BP: trichoblast differentiation (GO:0010054); BP: glucuronoxylan metabolic process (GO:0010413); MF: glucuronoxylan 4-O-methyltransferase activity (GO:0030775); BP: xylan biosynthetic process (GO:0045492); | -- |
| BnaA10g10690D | 3.5413288 | 3.55E-13 | up | MF: ATP transmembrane transporter activity (GO:0005347); CC: mitochondrial inner membrane (GO:0005743); BP: mitochondrial transport (GO:0006839); BP: anther development (GO:0048653); | -- |
| BnaA10g14780D | -2.129824 | 0.000196339 | down | MF: beta-galactosidase activity (GO:0004565); BP: carbohydrate metabolic process (GO:0005975); MF: carbohydrate binding (GO:0030246); MF: cation binding (GO:0043169); CC: apoplast (GO:0048046); | -- |
| BnaA10g30190D | -2.420887 | 4.90E-06 | down | CC: extracellular region (GO:0005576); MF: lipid binding (GO:0008289); CC: chloroplast (GO:0009507); BP: plant-type cell wall modification (GO:0009827); BP: pollen tube growth (GO:0009860); CC: monolayer-surrounded lipid storage body (GO:0012511); CC: integral component of membrane (GO:0016021); BP: lipid storage (GO:0019915); BP: sexual reproduction (GO:0019953); | -- |
| BnaAnng03750D | -2.698342 | 7.07E-10 | down | CC: Golgi apparatus (GO:0005794); BP: plant-type secondary cell wall biogenesis (GO:0009834); BP: glucuronoxylan metabolic process (GO:0010413); BP: xylan biosynthetic process (GO:0045492); | -- |
| BnaAnng13970D | -2.887069 | 1.34E-20 | down | MF: copper ion binding (GO:0005507); MF: L-ascorbate oxidase activity (GO:0008447); BP: plant-type secondary cell wall biogenesis (GO:0009834); BP: glucuronoxylan metabolic process (GO:0010413); BP: xylan biosynthetic process (GO:0045492); BP: lignin catabolic process (GO:0046274); CC: apoplast (GO:0048046); MF: hydroquinone:oxygen oxidoreductase activity (GO:0052716); BP: oxidation-reduction process (GO:0055114); | -- |
| BnaAnng14670D | -2.798687 | 5.77E-11 | down | MF: carboxylic ester hydrolase activity (GO:0004091); CC: extracellular region (GO:0005576); BP: protein targeting to vacuole (GO:0006623); BP: lipid metabolic process (GO:0006629); BP: membrane fusion (GO:0006944); BP: signal transduction (GO:0007165); BP: vesicle-mediated transport (GO:0016192); BP: endosomal transport (GO:0016197); MF: lipase activity (GO:0016298); MF: phosphatidylinositol binding (GO:0035091); MF: metal ion binding (GO:0046872); | -- |
| BnaAnng17190D | 4.3825207 | 1.89E-22 | up | BP: maltose metabolic process (GO:0000023); BP: sucrose metabolic process (GO:0005985); BP: fructose metabolic process (GO:0006000); BP: pentose-phosphate shunt (GO:0006098); BP: rRNA processing (GO:0006364); BP: response to cold (GO:0009409); CC: chloroplast stroma (GO:0009570); BP: response to blue light (GO:0009637); BP: photosynthetic electron transport in photosystem I (GO:0009773); BP: chloroplast relocation (GO:0009902); BP: thylakoid membrane organization (GO:0010027); BP: response to red light (GO:0010114); BP: photosystem II assembly (GO:0010207); BP: response to far red light (GO:0010218); CC: stromule (GO:0010319); BP: chlorophyll biosynthetic process (GO:0015995); BP: carotenoid biosynthetic process (GO:0016117); BP: starch biosynthetic process (GO:0019252); BP: reductive pentose-phosphate cycle (GO:0019253); BP: cellular cation homeostasis (GO:0030003); BP: fructose 1,6-bisphosphate metabolic process (GO:0030388); BP: regulation of protein dephosphorylation (GO:0035304); MF: fructose 1,6-bisphosphate 1-phosphatase activity (GO:0042132); BP: defense response to bacterium (GO:0042742); BP: positive regulation of catalytic activity (GO:0043085); MF: metal ion binding (GO:0046872); CC: apoplast (GO:0048046); BP: divalent metal ion transport (GO:0070838); | Glycolysis / Gluconeogenesis (ko00010); Pentose phosphate pathway (ko00030); Fructose and mannose metabolism (ko00051); Carbon fixation in photosynthetic organisms (ko00710); Carbon metabolism (ko01200) |
| BnaAnng18460D | -3.225975 | 7.11E-30 | down | CC: plasma membrane (GO:0005886); BP: plant-type secondary cell wall biogenesis (GO:0009834); CC: anchored component of membrane (GO:0031225); | -- |
| BnaAnng19640D | 3.7830208 | 2.02E-15 | up | -- | -- |
| BnaAnng30250D | 2.3627977 | 2.76E-09 | up | -- | -- |
| BnaAnng30260D | 3.1679716 | 4.58E-20 | up | MF: transcription factor activity, sequence-specific DNA binding (GO:0003700); CC: nucleus (GO:0005634); BP: response to xenobiotic stimulus (GO:0009410); BP: response to ethylene (GO:0009723); BP: hormone-mediated signaling pathway (GO:0009755); BP: endoplasmic reticulum unfolded protein response (GO:0030968); BP: positive regulation of transcription, DNA-templated (GO:0045893); MF: protein heterodimerization activity (GO:0046982); BP: positive regulation of seed maturation (GO:2000693); | -- |
| BnaAnng35110D | -2.218327 | 5.90E-05 | down | CC: chloroplast (GO:0009507); BP: embryo development ending in seed dormancy (GO:0009793); BP: pollen exine formation (GO:0010584); | -- |
| BnaAnng37210D | -3.46371 | 1.75E-15 | down | CC: extracellular region (GO:0005576); BP: lipid metabolic process (GO:0006629); MF: lipase activity (GO:0016298); | -- |
| BnaC01g04850D | -2.518221 | 6.56E-08 | down | CC: Golgi apparatus (GO:0005794); BP: starch metabolic process (GO:0005982); BP: plant-type secondary cell wall biogenesis (GO:0009834); BP: meristem initiation (GO:0010014); BP: xylem development (GO:0010089); BP: glucuronoxylan biosynthetic process (GO:0010417); MF: glucuronoxylan glucuronosyltransferase activity (GO:0080116); | -- |
| BnaC01g27600D | 2.1658366 | 4.68E-05 | up | MF: nucleotide binding (GO:0000166); MF: protease binding (GO:0002020); CC: cell wall (GO:0005618); CC: nucleolus (GO:0005730); CC: vacuolar membrane (GO:0005774); CC: Golgi apparatus (GO:0005794); CC: plasma membrane (GO:0005886); BP: gluconeogenesis (GO:0006094); BP: glycolytic process (GO:0006096); BP: protein folding (GO:0006457); BP: response to heat (GO:0009408); BP: response to cold (GO:0009409); CC: plasmodesma (GO:0009506); CC: chloroplast (GO:0009507); BP: response to virus (GO:0009615); BP: response to salt stress (GO:0009651); BP: negative regulation of seed germination (GO:0010187); CC: cytosolic ribosome (GO:0022626); BP: defense response to bacterium (GO:0042742); BP: response to cadmium ion (GO:0046686); CC: apoplast (GO:0048046); BP: defense response to fungus (GO:0050832); BP: stomatal closure (GO:0090332); | Spliceosome (ko03040); Protein processing in endoplasmic reticulum (ko04141); Endocytosis (ko04144) |
| BnaC01g31750D | -2.061833 | 0.000404275 | down | CC: extracellular region (GO:0005576); | -- |
| BnaC01g36080D | -3.443785 | 2.96E-14 | down | BP: protein glycosylation (GO:0006486); MF: Ran GTPase binding (GO:0008536); | -- |
| BnaC01g43270D | 2.6073027 | 3.86E-07 | up | MF: magnesium ion binding (GO:0000287); MF: adenosylmethionine-8-amino-7-oxononanoate transaminase activity (GO:0004015); MF: dethiobiotin synthase activity (GO:0004141); MF: ATP binding (GO:0005524); CC: mitochondrion (GO:0005739); BP: DNA replication initiation (GO:0006270); BP: regulation of DNA replication (GO:0006275); BP: DNA methylation (GO:0006306); BP: cell proliferation (GO:0008283); BP: biotin biosynthetic process (GO:0009102); MF: pyridoxal phosphate binding (GO:0030170); BP: histone H3-K9 methylation (GO:0051567); BP: regulation of cell cycle (GO:0051726); | Biotin metabolism (ko00780) |
| BnaC01g43920D | -2.761386 | 5.49E-14 | down | CC: nucleus (GO:0005634); CC: Golgi apparatus (GO:0005794); BP: starch metabolic process (GO:0005982); BP: plant-type secondary cell wall biogenesis (GO:0009834); BP: glucuronoxylan biosynthetic process (GO:0010417); MF: glucuronoxylan glucuronosyltransferase activity (GO:0080116); | -- |
| BnaC02g05120D | 4.6278428 | 7.89E-26 | up | CC: cell wall (GO:0005618); CC: mitochondrial inner membrane (GO:0005743); CC: vacuolar membrane (GO:0005774); CC: Golgi apparatus (GO:0005794); BP: pentose-phosphate shunt (GO:0006098); BP: transport (GO:0006810); CC: chloroplast (GO:0009507); BP: response to salt stress (GO:0009651); CC: integral component of membrane (GO:0016021); | -- |
| BnaC02g16120D | -2.01563 | 0.000322157 | down | MF: copper ion binding (GO:0005507); CC: extracellular region (GO:0005576); CC: plant-type cell wall (GO:0009505); CC: plasmodesma (GO:0009506); MF: oxidoreductase activity (GO:0016491); BP: oxidation-reduction process (GO:0055114); | -- |
| BnaC02g16970D | -2.766084 | 1.43E-13 | down | CC: extracellular region (GO:0005576); BP: lipid transport (GO:0006869); MF: lipid binding (GO:0008289); BP: pollen exine formation (GO:0010584); | -- |
| BnaC02g23340D | -2.516564 | 1.53E-07 | down | MF: carboxylic ester hydrolase activity (GO:0004091); CC: extracellular region (GO:0005576); CC: chloroplast (GO:0009507); BP: pollen exine formation (GO:0010584); MF: lipase activity (GO:0016298); MF: transferase activity, transferring acyl groups (GO:0016746); BP: sexual reproduction (GO:0019953); BP: primary metabolic process (GO:0044238); BP: organic substance metabolic process (GO:0071704); | -- |
| BnaC02g23350D | -2.302573 | 8.40E-05 | down | MF: carboxylic ester hydrolase activity (GO:0004091); CC: extracellular region (GO:0005576); BP: lipid metabolic process (GO:0006629); CC: plasmodesma (GO:0009506); CC: chloroplast (GO:0009507); BP: pollen exine formation (GO:0010584); CC: membrane (GO:0016020); MF: lipase activity (GO:0016298); MF: transferase activity, transferring acyl groups (GO:0016746); BP: glucosinolate biosynthetic process (GO:0019761); BP: sexual reproduction (GO:0019953); BP: defense response to fungus (GO:0050832); | -- |
| BnaC02g36760D | -3.111753 | 4.11E-12 | down | MF: copper ion binding (GO:0005507); CC: plasma membrane (GO:0005886); MF: electron carrier activity (GO:0009055); BP: glucuronoxylan metabolic process (GO:0010413); CC: anchored component of membrane (GO:0031225); BP: xylan biosynthetic process (GO:0045492); | -- |
| BnaC03g03150D | -2.886444 | 1.10E-10 | down | CC: extracellular region (GO:0005576); MF: lipid binding (GO:0008289); CC: chloroplast (GO:0009507); BP: plant-type cell wall modification (GO:0009827); BP: pollen tube growth (GO:0009860); CC: monolayer-surrounded lipid storage body (GO:0012511); CC: integral component of membrane (GO:0016021); BP: lipid storage (GO:0019915); BP: sexual reproduction (GO:0019953); | -- |
| BnaC03g42480D | -2.893041 | 5.01E-08 | down | -- | -- |
| BnaC03g57080D | 2.7622218 | 1.94E-10 | up | CC: chloroplast (GO:0009507); | -- |
| BnaC03g57690D | -2.114503 | 7.52E-05 | down | CC: Golgi apparatus (GO:0005794); BP: plant-type secondary cell wall biogenesis (GO:0009834); BP: glucuronoxylan biosynthetic process (GO:0010417); CC: membrane (GO:0016020); MF: glucuronoxylan glucuronosyltransferase activity (GO:0080116); | -- |
| BnaC03g65980D | -2.593718 | 1.71E-06 | down | BP: fatty acid biosynthetic process (GO:0006633); CC: membrane (GO:0016020); MF: transferase activity, transferring acyl groups other than amino-acyl groups (GO:0016747); | Fatty acid elongation (ko00062) |
| BnaC04g00270D | -2.376702 | 1.83E-06 | down | CC: mitochondrion (GO:0005739); | -- |
| BnaC04g06070D | 3.3164431 | 3.98E-11 | up | -- | -- |
| BnaC04g07220D | -2.369736 | 9.51E-10 | down | MF: copper ion binding (GO:0005507); BP: lignin biosynthetic process (GO:0009809); BP: plant-type secondary cell wall biogenesis (GO:0009834); BP: glucuronoxylan metabolic process (GO:0010413); BP: xylan biosynthetic process (GO:0045492); BP: lignin catabolic process (GO:0046274); CC: apoplast (GO:0048046); MF: hydroquinone:oxygen oxidoreductase activity (GO:0052716); BP: oxidation-reduction process (GO:0055114); | -- |
| BnaC05g15620D | -4.582562 | 4.23E-25 | down | BP: lipid metabolic process (GO:0006629); MF: lipase activity (GO:0016298); | -- |
| BnaC05g15630D | -2.736594 | 2.60E-09 | down | MF: carboxylic ester hydrolase activity (GO:0004091); CC: extracellular region (GO:0005576); BP: lipid metabolic process (GO:0006629); MF: lipase activity (GO:0016298); | -- |
| BnaC05g28280D | -2.229893 | 0.000198599 | down | CC: Golgi apparatus (GO:0005794); BP: protein glycosylation (GO:0006486); BP: pollen exine formation (GO:0010584); CC: integral component of membrane (GO:0016021); MF: galactosylxylosylprotein 3-beta-galactosyltransferase activity (GO:0047220); | -- |
| BnaC06g01060D | -3.443154 | 8.54E-19 | down | MF: peroxidase activity (GO:0004601); CC: extracellular region (GO:0005576); BP: response to oxidative stress (GO:0006979); MF: heme binding (GO:0020037); MF: metal ion binding (GO:0046872); BP: oxidation-reduction process (GO:0055114); | Phenylpropanoid biosynthesis (ko00940) |
| BnaC06g07780D | -2.733061 | 1.62E-21 | down | CC: extracellular region (GO:0005576); CC: mitochondrion (GO:0005739); CC: Golgi apparatus (GO:0005794); BP: lignin metabolic process (GO:0009808); BP: trichoblast differentiation (GO:0010054); BP: glucuronoxylan metabolic process (GO:0010413); MF: glucuronoxylan 4-O-methyltransferase activity (GO:0030775); BP: xylan biosynthetic process (GO:0045492); | -- |
| BnaC06g15710D | -2.751843 | 1.43E-15 | down | CC: nucleus (GO:0005634); BP: response to water deprivation (GO:0009414); BP: response to salt stress (GO:0009651); BP: positive regulation of transcription, DNA-templated (GO:0045893); BP: response to freezing (GO:0050826); | -- |
| BnaC06g16950D | 3.1415119 | 5.20E-15 | up | -- | -- |
| BnaC06g22910D | -3.032359 | 4.51E-11 | down | MF: iron ion binding (GO:0005506); CC: mitochondrion (GO:0005739); BP: spermidine metabolic process (GO:0008216); MF: electron carrier activity (GO:0009055); BP: pollen exine formation (GO:0010584); MF: oxygen binding (GO:0019825); MF: heme binding (GO:0020037); BP: oxidation-reduction process (GO:0055114); MF: tricoumaroylspermidine meta-hydroxylase activity (GO:0072547); MF: dicoumaroyl monocaffeoyl spermidine meta-hydroxylase activity (GO:0072548); MF: monocoumaroyl dicaffeoyl spermidine meta-hydroxylase activity (GO:0072549); MF: triferuloylspermidine meta-hydroxylase activity (GO:0072550); MF: diferuloyl mono-(hydroxyferuloyl) spermidine meta-hydroxylase activity (GO:0072551); MF: monoferuloyl di-(hydroxyferuloyl) spermidine meta-hydroxylase activity (GO:0072552); | Phenylpropanoid biosynthesis (ko00940) |
| BnaC06g27820D | -2.063393 | 7.58E-09 | down | CC: extracellular region (GO:0005576); BP: lipid transport (GO:0006869); MF: lipid binding (GO:0008289); BP: pollen exine formation (GO:0010584); | -- |
| BnaC06g30350D | -2.481412 | 1.53E-14 | down | CC: plasma membrane (GO:0005886); CC: anchored component of membrane (GO:0031225); | -- |
| BnaC06g30570D | 2.5872404 | 2.07E-07 | up | MF: translation initiation factor activity (GO:0003743); MF: translation elongation factor activity (GO:0003746); CC: cytoplasm (GO:0005737); BP: translational initiation (GO:0006413); BP: translational frameshifting (GO:0006452); BP: peptidyl-lysine modification to peptidyl-hypusine (GO:0008612); BP: xylem development (GO:0010089); MF: ribosome binding (GO:0043022); BP: positive regulation of translational elongation (GO:0045901); BP: positive regulation of translational termination (GO:0045905); | -- |
| BnaC06g41610D | 2.8573907 | 1.52E-08 | up | -- | -- |
| BnaC07g17610D | -2.301982 | 3.39E-11 | down | CC: plasma membrane (GO:0005886); MF: zinc ion binding (GO:0008270); BP: plant-type secondary cell wall biogenesis (GO:0009834); BP: salicylic acid mediated signaling pathway (GO:0009863); BP: jasmonic acid mediated signaling pathway (GO:0009867); BP: ethylene-activated signaling pathway (GO:0009873); BP: glucuronoxylan metabolic process (GO:0010413); CC: integral component of membrane (GO:0016021); MF: cellulose synthase (UDP-forming) activity (GO:0016760); BP: cellulose biosynthetic process (GO:0030244); BP: defense response to bacterium (GO:0042742); BP: xylan biosynthetic process (GO:0045492); BP: defense response to fungus (GO:0050832); BP: cell wall thickening (GO:0052386); | -- |
| BnaC07g24070D | -3.263634 | 6.25E-24 | down | MF: copper ion binding (GO:0005507); CC: plasma membrane (GO:0005886); MF: electron carrier activity (GO:0009055); BP: glucuronoxylan metabolic process (GO:0010413); CC: anchored component of membrane (GO:0031225); BP: xylan biosynthetic process (GO:0045492); | -- |
| BnaC07g31360D | -2.244714 | 4.65E-05 | down | -- | -- |
| BnaC07g44200D | -4.092137 | 3.11E-23 | down | -- | -- |
| BnaC07g50180D | -2.433873 | 2.03E-10 | down | CC: cell wall (GO:0005618); CC: plasma membrane (GO:0005886); BP: response to osmotic stress (GO:0006970); BP: response to water deprivation (GO:0009414); BP: plant-type secondary cell wall biogenesis (GO:0009834); BP: salicylic acid mediated signaling pathway (GO:0009863); BP: jasmonic acid mediated signaling pathway (GO:0009867); BP: ethylene-activated signaling pathway (GO:0009873); BP: positive regulation of abscisic acid biosynthetic process (GO:0010116); BP: glucuronoxylan metabolic process (GO:0010413); CC: integral component of membrane (GO:0016021); MF: cellulose synthase (UDP-forming) activity (GO:0016760); BP: cellulose biosynthetic process (GO:0030244); BP: defense response to bacterium (GO:0042742); BP: xylan biosynthetic process (GO:0045492); BP: defense response to fungus (GO:0050832); BP: cell wall thickening (GO:0052386); | -- |
| BnaC08g01470D | 2.6972768 | 1.51E-07 | up | CC: plasma membrane (GO:0005886); BP: oligopeptide transport (GO:0006857); MF: kinase activity (GO:0016301); | -- |
| BnaC08g01740D | -2.722079 | 1.04E-08 | down | BP: proteolysis (GO:0006508); MF: cysteine-type peptidase activity (GO:0008234); MF: oxidoreductase activity (GO:0016491); CC: apoplast (GO:0048046); | -- |
| BnaC08g21610D | -2.158539 | 5.26E-05 | down | CC: Golgi apparatus (GO:0005794); BP: plant-type secondary cell wall biogenesis (GO:0009834); BP: glucuronoxylan metabolic process (GO:0010413); BP: xylan biosynthetic process (GO:0045492); | -- |
| BnaC08g35720D | 5.9155064 | 2.32E-43 | up | CC: vacuolar proton-transporting V-type ATPase, V0 domain (GO:0000220); CC: mitochondrion (GO:0005739); CC: Golgi apparatus (GO:0005794); BP: obsolete ATP catabolic process (GO:0006200); CC: chloroplast (GO:0009507); MF: hydrogen-translocating pyrophosphatase activity (GO:0009678); CC: plant-type vacuole membrane (GO:0009705); MF: hydrogen ion transmembrane transporter activity (GO:0015078); BP: ATP synthesis coupled proton transport (GO:0015986); BP: ATP hydrolysis coupled proton transport (GO:0015991); MF: ATPase activity (GO:0016887); BP: cellular response to nutrient levels (GO:0031669); BP: sequestering of zinc ion (GO:0032119); BP: vacuolar sequestering (GO:0043181); MF: nutrient reservoir activity (GO:0045735); BP: vacuolar proton-transporting V-type ATPase complex assembly (GO:0070072); BP: cellular response to salt stress (GO:0071472); | Oxidative phosphorylation (ko00190); Phagosome (ko04145) |
| BnaC08g36010D | 2.0209828 | 0.000465268 | up | CC: nucleus (GO:0005634); BP: response to blue light (GO:0009637); BP: embryo development ending in seed dormancy (GO:0009793); BP: seed germination (GO:0009845); BP: ethylene-activated signaling pathway (GO:0009873); BP: regulation of flower development (GO:0009909); BP: meristem structural organization (GO:0009933); BP: regulation of photomorphogenesis (GO:0010099); BP: response to red light (GO:0010114); BP: seed dormancy process (GO:0010162); BP: sugar mediated signaling pathway (GO:0010182); BP: vegetative to reproductive phase transition of meristem (GO:0010228); BP: protein ubiquitination (GO:0016567); BP: lipid storage (GO:0019915); BP: regulation of circadian rhythm (GO:0042752); BP: response to freezing (GO:0050826); | -- |
| BnaC08g36360D | 2.6055252 | 6.82E-08 | up | MF: nucleotide binding (GO:0000166); MF: catalytic activity (GO:0003824); CC: mitochondrial respiratory chain complex I (GO:0005747); BP: ubiquitin-dependent protein catabolic process (GO:0006511); BP: response to salt stress (GO:0009651); BP: photorespiration (GO:0009853); MF: coenzyme binding (GO:0050662); BP: response to misfolded protein (GO:0051788); BP: proteasome core complex assembly (GO:0080129); | Oxidative phosphorylation (ko00190) |
| BnaC08g36570D | 5.6518351 | 4.06E-44 | up | MF: actin binding (GO:0003779); CC: cell wall (GO:0005618); CC: nucleolus (GO:0005730); CC: spindle (GO:0005819); CC: cytosol (GO:0005829); CC: plasma membrane (GO:0005886); BP: actin polymerization or depolymerization (GO:0008154); CC: plasmodesma (GO:0009506); CC: chloroplast (GO:0009507); CC: phragmoplast (GO:0009524); BP: unidimensional cell growth (GO:0009826); CC: actin cytoskeleton (GO:0015629); CC: apoplast (GO:0048046); | -- |
| BnaC08g38630D | 2.6562621 | 4.52E-08 | up | CC: nucleus (GO:0005634); | -- |
| BnaC08g39040D | 3.7131958 | 1.01E-14 | up | MF: CDP-diacylglycerol-serine O-phosphatidyltransferase activity (GO:0003882); CC: nucleus (GO:0005634); CC: mitochondrion (GO:0005739); CC: endoplasmic reticulum membrane (GO:0005789); BP: phosphatidylserine biosynthetic process (GO:0006659); | Glycerophospholipid metabolism (ko00564) |
| BnaC08g39110D | 3.6211435 | 2.97E-20 | up | MF: serine-type carboxypeptidase activity (GO:0004185); CC: extracellular region (GO:0005576); CC: vacuole (GO:0005773); BP: proteolysis (GO:0006508); | -- |
| BnaC08g39120D | 3.54706 | 3.92E-13 | up | -- | -- |
| BnaC08g39130D | 7.8635337 | 7.19E-100 | up | MF: copper ion binding (GO:0005507); MF: calmodulin binding (GO:0005516); MF: ATP binding (GO:0005524); CC: mitochondrion (GO:0005739); CC: cytosol (GO:0005829); BP: gluconeogenesis (GO:0006094); BP: glycolytic process (GO:0006096); BP: protein folding (GO:0006457); BP: tryptophan catabolic process (GO:0006569); BP: response to heat (GO:0009408); BP: response to cold (GO:0009409); CC: chloroplast thylakoid membrane (GO:0009535); CC: chloroplast stroma (GO:0009570); BP: response to high light intensity (GO:0009644); BP: response to salt stress (GO:0009651); BP: chloroplast organization (GO:0009658); BP: indoleacetic acid biosynthetic process (GO:0009684); CC: chloroplast envelope (GO:0009941); BP: isopentenyl diphosphate biosynthetic process, methylerythritol 4-phosphate pathway (GO:0019288); BP: cysteine biosynthetic process (GO:0019344); BP: response to endoplasmic reticulum stress (GO:0034976); BP: response to hydrogen peroxide (GO:0042542); BP: response to cadmium ion (GO:0046686); CC: apoplast (GO:0048046); BP: plant ovule development (GO:0048481); MF: chaperone binding (GO:0051087); BP: positive regulation of superoxide dismutase activity (GO:1901671); | -- |
| BnaC08g39360D | 4.3093829 | 1.76E-20 | up | MF: hydrolase activity, hydrolyzing O-glycosyl compounds (GO:0004553); CC: cell wall (GO:0005618); BP: cellular glucan metabolic process (GO:0006073); BP: phloem or xylem histogenesis (GO:0010087); BP: fruit development (GO:0010154); MF: xyloglucan:xyloglucosyl transferase activity (GO:0016762); CC: apoplast (GO:0048046); BP: stamen filament development (GO:0080086); | -- |
| BnaC08g39400D | 2.8664292 | 1.07E-13 | up | MF: DNA binding (GO:0003677); MF: transcription factor activity, sequence-specific DNA binding (GO:0003700); CC: nucleus (GO:0005634); BP: regulation of transcription, DNA-templated (GO:0006355); BP: response to ethylene (GO:0009723); BP: regulation of developmental process (GO:0050793); | -- |
| BnaC08g40400D | 2.8442221 | 6.78E-09 | up | MF: DNA binding (GO:0003677); CC: nucleus (GO:0005634); CC: Golgi apparatus (GO:0005794); CC: cytosol (GO:0005829); BP: glucose catabolic process (GO:0006007); MF: GTPase activator activity (GO:0008060); MF: zinc ion binding (GO:0008270); BP: cellulose biosynthetic process (GO:0030244); BP: regulation of GTPase activity (GO:0032312); BP: Golgi vesicle transport (GO:0048193); | Endocytosis (ko04144) |
| BnaC08g40410D | 5.1073013 | 1.89E-33 | up | MF: GTPase activator activity (GO:0005098); CC: nuclear envelope (GO:0005635); CC: vacuolar membrane (GO:0005774); CC: endoplasmic reticulum (GO:0005783); BP: nucleocytoplasmic transport (GO:0006913); BP: toxin catabolic process (GO:0009407); CC: chloroplast (GO:0009507); BP: photomorphogenesis (GO:0009640); BP: response to salt stress (GO:0009651); BP: protein deneddylation (GO:0010388); BP: lateral root development (GO:0048527); | RNA transport (ko03013) |
| BnaC08g40810D | 2.1656638 | 1.34E-12 | up | MF: protein serine/threonine kinase activity (GO:0004674); BP: protein autophosphorylation (GO:0046777); | -- |
| BnaC09g05400D | 4.1128235 | 4.72E-19 | up | CC: nucleus (GO:0005634); | -- |
| BnaC09g05590D | 2.3524155 | 6.78E-09 | up | MF: pectinesterase activity (GO:0030599); BP: negative regulation of catalytic activity (GO:0043086); MF: pectinesterase inhibitor activity (GO:0046910); | -- |
| BnaC09g05960D | 4.2660415 | 2.25E-32 | up | MF: DNA binding (GO:0003677); CC: nucleus (GO:0005634); | -- |
| BnaC09g06220D | 3.6145778 | 1.94E-17 | up | CC: plasma membrane (GO:0005886); CC: chloroplast (GO:0009507); | -- |
| BnaC09g06260D | 5.1503158 | 1.38E-34 | up | CC: nucleus (GO:0005634); | -- |
| BnaC09g31020D | -2.574052 | 1.64E-12 | down | CC: Golgi apparatus (GO:0005794); BP: plant-type cell wall biogenesis (GO:0009832); BP: xylem development (GO:0010089); BP: glucuronoxylan biosynthetic process (GO:0010417); MF: polygalacturonate 4-alpha-galacturonosyltransferase activity (GO:0047262); BP: cell wall organization (GO:0071555); | Starch and sucrose metabolism (ko00500); Amino sugar and nucleotide sugar metabolism (ko00520) |
| BnaC09g53990D | 4.7146429 | 1.37E-27 | up | BP: sulfur amino acid metabolic process (GO:0000096); BP: MAPK cascade (GO:0000165); MF: iron ion binding (GO:0005506); CC: nucleus (GO:0005634); BP: pentose-phosphate shunt (GO:0006098); BP: regulation of translation (GO:0006417); BP: glycine catabolic process (GO:0006546); BP: protein targeting to membrane (GO:0006612); BP: unsaturated fatty acid biosynthetic process (GO:0006636); BP: phosphatidylglycerol biosynthetic process (GO:0006655); BP: vitamin metabolic process (GO:0006766); BP: cellular amino acid biosynthetic process (GO:0008652); BP: aromatic amino acid family metabolic process (GO:0009072); BP: lipoate metabolic process (GO:0009106); BP: coenzyme biosynthetic process (GO:0009108); BP: response to cold (GO:0009409); CC: chloroplast stroma (GO:0009570); BP: detection of biotic stimulus (GO:0009595); BP: response to blue light (GO:0009637); BP: response to high light intensity (GO:0009644); BP: plastid organization (GO:0009657); BP: jasmonic acid biosynthetic process (GO:0009695); BP: salicylic acid biosynthetic process (GO:0009697); BP: response to sucrose (GO:0009744); BP: systemic acquired resistance, salicylic acid mediated signaling pathway (GO:0009862); BP: jasmonic acid mediated signaling pathway (GO:0009867); CC: chloroplast envelope (GO:0009941); BP: response to red light (GO:0010114); BP: regulation of proton transport (GO:0010155); BP: response to chitin (GO:0010200); BP: response to far red light (GO:0010218); BP: PSII associated light-harvesting complex II catabolic process (GO:0010304); BP: regulation of hydrogen peroxide metabolic process (GO:0010310); BP: regulation of plant-type hypersensitive response (GO:0010363); BP: chlorophyll biosynthetic process (GO:0015995); BP: carotenoid biosynthetic process (GO:0016117); BP: regulation of lipid metabolic process (GO:0019216); BP: starch biosynthetic process (GO:0019252); BP: isopentenyl diphosphate biosynthetic process, methylerythritol 4-phosphate pathway (GO:0019288); BP: photosynthesis, light reaction (GO:0019684); BP: glucosinolate metabolic process (GO:0019760); BP: negative regulation of defense response (GO:0031348); BP: oxylipin biosynthetic process (GO:0031408); BP: defense response to bacterium (GO:0042742); BP: regulation of multi-organism process (GO:0043900); BP: sulfur compound biosynthetic process (GO:0044272); BP: positive regulation of transcription, DNA-templated (GO:0045893); MF: 4-hydroxy-3-methylbut-2-en-1-yl diphosphate synthase activity (GO:0046429); BP: defense response to fungus (GO:0050832); MF: 4 iron, 4 sulfur cluster binding (GO:0051539); | Terpenoid backbone biosynthesis (ko00900) |
| BnaCnng07250D | -2.324309 | 2.54E-05 | down | -- | -- |
| BnaCnng08170D | -2.561603 | 3.08E-06 | down | CC: extracellular region (GO:0005576); BP: response to other organism (GO:0051707); | -- |
| BnaCnng17490D | 3.1193101 | 1.34E-20 | up | -- | -- |
| BnaCnng20520D | -2.040195 | 0.000289307 | down | MF: ATP binding (GO:0005524); CC: nucleus (GO:0005634); CC: plasma membrane (GO:0005886); BP: obsolete ATP catabolic process (GO:0006200); CC: plasmodesma (GO:0009506); CC: integral component of membrane (GO:0016021); MF: ATPase activity, coupled to transmembrane movement of substances (GO:0042626); BP: transmembrane transport (GO:0055085); | ABC transporters (ko02010) |
| BnaCnng24040D | 3.1264402 | 8.37E-11 | up | MF: protein binding (GO:0005515); CC: cytosol (GO:0005829); BP: glycolytic process (GO:0006096); BP: tricarboxylic acid cycle (GO:0006099); BP: iron ion transport (GO:0006826); BP: water transport (GO:0006833); BP: hyperosmotic response (GO:0006972); BP: Golgi organization (GO:0007030); MF: phosphoenolpyruvate carboxylase activity (GO:0008964); BP: response to temperature stimulus (GO:0009266); BP: response to salt stress (GO:0009651); BP: response to sucrose (GO:0009744); BP: response to glucose (GO:0009749); BP: response to fructose (GO:0009750); BP: cellular response to iron ion starvation (GO:0010106); BP: response to nitrate (GO:0010167); BP: nitrate transport (GO:0015706); BP: carbon fixation (GO:0015977); BP: cellular response to phosphate starvation (GO:0016036); BP: response to cadmium ion (GO:0046686); CC: apoplast (GO:0048046); BP: protein tetramerization (GO:0051262); | Pyruvate metabolism (ko00620); Carbon fixation in photosynthetic organisms (ko00710); Carbon metabolism (ko01200) |
| BnaCnng36050D | 2.3157515 | 3.21E-05 | up | -- | -- |
| BnaCnng36380D | -2.533736 | 5.39E-08 | down | CC: extracellular region (GO:0005576); MF: lipid binding (GO:0008289); CC: chloroplast (GO:0009507); BP: plant-type cell wall modification (GO:0009827); BP: pollen tube growth (GO:0009860); CC: monolayer-surrounded lipid storage body (GO:0012511); CC: integral component of membrane (GO:0016021); BP: lipid storage (GO:0019915); BP: sexual reproduction (GO:0019953); | -- |
| BnaCnng39540D | -2.105265 | 2.34E-06 | down | MF: cysteine-type endopeptidase inhibitor activity (GO:0004869); CC: extracellular region (GO:0005576); BP: fatty acid catabolic process (GO:0009062); BP: seed dormancy process (GO:0010162); BP: negative regulation of peptidase activity (GO:0010466); | -- |
| BnaCnng55000D | -3.062561 | 2.19E-14 | down | MF: carboxylic ester hydrolase activity (GO:0004091); CC: extracellular region (GO:0005576); BP: lipid metabolic process (GO:0006629); MF: lipase activity (GO:0016298); | -- |
| BnaCnng60800D | -3.475119 | 4.82E-13 | down | CC: extracellular region (GO:0005576); BP: lipid transport (GO:0006869); MF: lipid binding (GO:0008289); | -- |
| BnaCnng69820D | -2.010464 | 0.000343974 | down | MF: transporter activity (GO:0005215); CC: plasma membrane (GO:0005886); BP: oligopeptide transport (GO:0006857); BP: response to nematode (GO:0009624); CC: integral component of membrane (GO:0016021); | -- |
| Brassica_napus_newGene_10149 | 2.2527154 | 5.04E-08 | up | -- | -- |
| Brassica_napus_newGene_10523 | 2.0363565 | 3.43E-11 | up | -- | -- |
| Brassica_napus_newGene_11035 | 3.9752785 | 1.52E-17 | up | MF: hydrolase activity, acting on ester bonds (GO:0016788); | -- |
| Brassica_napus_newGene_12095 | 4.8078517 | 2.87E-28 | up | CC: cell wall (GO:0005618); CC: vacuole (GO:0005773); CC: endoplasmic reticulum (GO:0005783); CC: plasma membrane (GO:0005886); CC: plasmodesma (GO:0009506); CC: integral component of membrane (GO:0016021); MF: transmembrane transporter activity (GO:0022857); BP: transmembrane transport (GO:0055085); | -- |
| Brassica_napus_newGene_12198 | 2.0160254 | 1.21E-05 | up | -- | -- |
| Brassica_napus_newGene_1404 | -3.474581 | 1.76E-13 | down | MF: transmembrane receptor protein serine/threonine kinase activity (GO:0004675); MF: ATP binding (GO:0005524); CC: integral component of plasma membrane (GO:0005887); BP: cell surface receptor signaling pathway (GO:0007166); BP: response to wounding (GO:0009611); BP: response to fungus (GO:0009620); BP: protein autophosphorylation (GO:0046777); | -- |
| Brassica_napus_newGene_1427 | 2.8149409 | 1.87E-10 | up | -- | -- |
| Brassica_napus_newGene_1645 | 2.0139266 | 0.000165215 | up | CC: nucleus (GO:0005634); BP: response to water deprivation (GO:0009414); | -- |
| Brassica_napus_newGene_2419 | -2.561304 | 6.57E-07 | down | -- | -- |
| Brassica_napus_newGene_3308 | 6.5570779 | 4.73E-63 | up | MF: ATP binding (GO:0005524); CC: cell wall (GO:0005618); CC: mitochondrion (GO:0005739); CC: chloroplast stroma (GO:0009570); | Protein processing in endoplasmic reticulum (ko04141); Plant-pathogen interaction (ko04626) |
| Brassica_napus_newGene_3329 | 3.5151951 | 4.32E-25 | up | -- | -- |
| Brassica_napus_newGene_3915 | -2.044211 | 1.61E-06 | down | -- | -- |
| Brassica_napus_newGene_5291 | 2.2522734 | 7.14E-05 | up | -- | -- |
| Brassica_napus_newGene_8007 | 3.9248077 | 4.82E-17 | up | -- | -- |
| Brassica_napus_newGene_87 | 2.1584605 | 5.26E-05 | up | CC: integral component of membrane (GO:0016021); | -- |
