# Supplementary material for: Investigation for a multi-silique trait in Brassica napus by alternative splicing analysis
Source: PeerJ. 2020 Oct 8;8:e10135. doi: 10.7717/peerj.10135 (PMC7548069; doi:10.7717/peerj.10135)
Supplement: Supplemental Information 8 [file peerj-08-10135-s008.docx]

Table S7. The 205 genes with line-specific AS events and their annotations.

|  | Gene ID | KEGG annotation | GO annotation | FDR for FC | log_2_FC |
| --- | --- | --- | --- | --- | --- |
| 1 | BnaA01g01260D | -- | Cellular Component: intracellular (GO:0005622); Biological Process: transport (GO:0006810); Molecular Function: phosphatidylinositol transporter activity (GO:0008526); | -- | -- |
| 2 | BnaA01g02000D | -- | Molecular Function: calmodulin binding (GO:0005516); Cellular Component: endoplasmic reticulum (GO:0005783); Biological Process: tRNA modification (GO:0006400); Biological Process: meristem structural organization (GO:0009933); Molecular Function: purine nucleotide binding (GO:0017076); Molecular Function: dihydrokaempferol 4-reductase activity (GO:0045552); Biological Process: seed development (GO:0048316); Biological Process: leaf development (GO:0048366); Molecular Function: coenzyme binding (GO:0050662); Biological Process: oxidation-reduction process (GO:0055114); Biological Process: sporopollenin biosynthetic process (GO:0080110); Biological Process: 5-carbamoylmethyluridine metabolic process (GO:0080178); | -- | -- |
| 3 | BnaA01g08850D | K14500\|0\|brp:103855933\|probable serine/threonine-protein kinase At4g35230; K14500 BR-signaling kinase [EC:2.7.11.1] (A) | Molecular Function: protein kinase activity (GO:0004672); Molecular Function: ATP binding (GO:0005524); Cellular Component: plasma membrane (GO:0005886); Biological Process: protein phosphorylation (GO:0006468); Biological Process: brassinosteroid mediated signaling pathway (GO:0009742); | 0.522231 | 1.436174 |
| 4 | BnaA01g10340D | -- | Cellular Component: mitochondrion (GO:0005739); Molecular Function: N-acetyltransferase activity (GO:0008080); Biological Process: metabolic process (GO:0008152); Biological Process: leaf morphogenesis (GO:0009965); Biological Process: cell differentiation (GO:0030154); | -0.20478 | 0.867671 |
| 5 | BnaA01g11490D | K12829\|0\|brp:103859260\|splicing factor 3B subunit 2; K12829 splicing factor 3B subunit 2 (A) | Cellular Component: nucleus (GO:0005634); Cellular Component: cytosol (GO:0005829); Biological Process: mRNA processing (GO:0006397); | -0.06171 | 0.95813 |
| 6 | BnaA01g14040D | -- | Biological Process: nuclear-transcribed mRNA catabolic process (GO:0000956); Biological Process: glucuronoxylan metabolic process (GO:0010413); Biological Process: regulation of gene expression, epigenetic (GO:0040029); Biological Process: xylan biosynthetic process (GO:0045492); | -0.05857 | 0.960215 |
| 7 | BnaA01g19500D | -- | Molecular Function: protein serine/threonine phosphatase activity (GO:0004722); Cellular Component: plasma membrane (GO:0005886); Biological Process: protein dephosphorylation (GO:0006470); Cellular Component: protein serine/threonine phosphatase complex (GO:0008287); Cellular Component: chloroplast (GO:0009507); Molecular Function: metal ion binding (GO:0046872); | 1.253769 | 2.384636 |
| 8 | BnaA01g21280D | -- | Cellular Component: mitochondrion (GO:0005739); | -0.42796 | 0.743314 |
| 9 | BnaA01g25360D | -- | Molecular Function: nucleotide binding (GO:0000166); Molecular Function: RNA binding (GO:0003723); Cellular Component: nucleus (GO:0005634); | 0.275901 | 1.21075 |
| 10 | BnaA01g26200D | K14432\|9.1103e-84\|brp:103837451\|ABSCISIC ACID-INSENSITIVE 5-like protein 7; K14432 ABA responsive element binding factor (A) | Molecular Function: sequence-specific DNA binding transcription factor activity (GO:0003700); Cellular Component: nucleus (GO:0005634); Biological Process: response to xenobiotic stimulus (GO:0009410); Biological Process: response to water deprivation (GO:0009414); Biological Process: response to mechanical stimulus (GO:0009612); Biological Process: response to salt stress (GO:0009651); Biological Process: abscisic acid-activated signaling pathway (GO:0009738); Biological Process: calcium-mediated signaling (GO:0019722); Biological Process: endoplasmic reticulum unfolded protein response (GO:0030968); Molecular Function: sequence-specific DNA binding (GO:0043565); Biological Process: positive regulation of transcription, DNA-templated (GO:0045893); Molecular Function: protein dimerization activity (GO:0046983); | -0.17491 | 0.885823 |
| 11 | BnaA02g01850D | -- | Molecular Function: sugar:proton symporter activity (GO:0005351); Cellular Component: plasma membrane (GO:0005886); Biological Process: response to nematode (GO:0009624); Cellular Component: plant-type vacuole membrane (GO:0009705); Biological Process: response to zinc ion (GO:0010043); Cellular Component: integral component of membrane (GO:0016021); Biological Process: carbohydrate transmembrane transport (GO:0034219); Biological Process: zinc ion homeostasis (GO:0055069); | -- | -- |
| 12 | BnaA02g03380D | K15425\|2.52712e-150\|brp:103851081\|serine/threonine-protein phosphatase 4 regulatory subunit 2; K15425 serine/threonine-protein phosphatase 4 regulatory subunit 2 (A) | Cellular Component: nucleus (GO:0005634); | 0.220711 | 1.165307 |
| 13 | BnaA02g22260D | K11673\|0\|brp:103848823\|actin-related protein 9; K11673 actin-related protein 8 (A) | Cellular Component: cytoplasm (GO:0005737); Biological Process: chromatin remodeling (GO:0006338); Biological Process: vegetative to reproductive phase transition of meristem (GO:0010228); Cellular Component: Ino80 complex (GO:0031011); | 0.07618 | 1.054223 |
| 14 | BnaA02g24780D | -- | Molecular Function: sequence-specific DNA binding transcription factor activity (GO:0003700); Cellular Component: nucleus (GO:0005634); Biological Process: regulation of transcription, DNA-templated (GO:0006355); Biological Process: response to chitin (GO:0010200); | -0.0361 | 0.975285 |
| 15 | BnaA02g27140D | K01623\|0\|brp:103832773\|probable fructose-bisphosphate aldolase 3, chloroplastic; K01623 fructose-bisphosphate aldolase, class I [EC:4.1.2.13] (A) | Molecular Function: fructose-bisphosphate aldolase activity (GO:0004332); Cellular Component: mitochondrion (GO:0005739); Biological Process: glycolytic process (GO:0006096); Biological Process: pentose-phosphate shunt (GO:0006098); Biological Process: response to oxidative stress (GO:0006979); Cellular Component: chloroplast thylakoid (GO:0009534); Biological Process: response to salt stress (GO:0009651); Cellular Component: plastoglobule (GO:0010287); Biological Process: response to cadmium ion (GO:0046686); | -0.07202 | 0.951307 |
| 16 | BnaA02g27520D | K00993\|0\|aly:ARALYDRAFT_484318\|hypothetical protein; K00993 ethanolaminephosphotransferase [EC:2.7.8.1] (A) | Cellular Component: Golgi apparatus (GO:0005794); Biological Process: phospholipid biosynthetic process (GO:0008654); Cellular Component: membrane (GO:0016020); Molecular Function: phosphatidyltransferase activity (GO:0030572); Biological Process: response to cadmium ion (GO:0046686); | -0.05395 | 0.963298 |
| 17 | BnaA02g36330D | -- | Cellular Component: nucleus (GO:0005634); | -- | -- |
| 18 | BnaA03g03350D | -- | Molecular Function: DNA binding (GO:0003677); Cellular Component: nucleus (GO:0005634); Biological Process: chromatin assembly or disassembly (GO:0006333); | -- | -- |
| 19 | BnaA03g04460D | K13366\|0\|brp:103856027\|polyamine oxidase 1; K13366 polyamine oxidase [EC:1.5.3.14 1.5.3.16 1.5.3.-] (A) | Cellular Component: mitochondrion (GO:0005739); Biological Process: polyamine catabolic process (GO:0006598); Molecular Function: polyamine oxidase activity (GO:0046592); Molecular Function: flavin adenine dinucleotide binding (GO:0050660); Biological Process: oxidation-reduction process (GO:0055114); | -0.27484 | 0.826544 |
| 20 | BnaA03g13540D | -- | Molecular Function: ATP binding (GO:0005524); Cellular Component: nucleus (GO:0005634); | -0.00793 | 0.994518 |
| 21 | BnaA03g13810D | -- | Cellular Component: plasma membrane (GO:0005886); Biological Process: potassium ion transport (GO:0006813); Biological Process: lithium ion transport (GO:0010351); Molecular Function: potassium ion transmembrane transporter activity (GO:0015079); Molecular Function: sodium:proton antiporter activity (GO:0015385); Cellular Component: integral component of membrane (GO:0016021); Biological Process: sodium ion transmembrane transport (GO:0035725); | 0.119874 | 1.08664 |
| 22 | BnaA03g15690D | -- | Molecular Function: protein binding (GO:0005515); Biological Process: leaf development (GO:0048366); | -- | -- |
| 23 | BnaA03g26620D | -- | -- | -- | -- |
| 24 | BnaA03g30380D | K11498\|0\|brp:103859267\|kinesin-related protein 4; K11498 centromeric protein E (A) | Molecular Function: microtubule motor activity (GO:0003777); Molecular Function: ATP binding (GO:0005524); Cellular Component: nucleus (GO:0005634); Cellular Component: kinesin complex (GO:0005871); Cellular Component: microtubule (GO:0005874); Biological Process: microtubule-based movement (GO:0007018); Biological Process: sister chromatid cohesion (GO:0007062); Molecular Function: microtubule binding (GO:0008017); Biological Process: chromatin silencing by small RNA (GO:0031048); Biological Process: meiotic chromosome segregation (GO:0045132); | 0.012188 | 1.008484 |
| 25 | BnaA03g39420D | K18752\|0\|brp:103860497\|transportin-1-like; K18752 transportin-1 (A) | Biological Process: protein import into nucleus, docking (GO:0000059); Cellular Component: nuclear pore (GO:0005643); Cellular Component: cytosol (GO:0005829); Biological Process: protein N-linked glycosylation (GO:0006487); Molecular Function: Ran GTPase binding (GO:0008536); Molecular Function: protein transporter activity (GO:0008565); Biological Process: multidimensional cell growth (GO:0009825); Biological Process: determination of bilateral symmetry (GO:0009855); Biological Process: radial pattern formation (GO:0009956); Biological Process: leaf morphogenesis (GO:0009965); Biological Process: meristem initiation (GO:0010014); Biological Process: meristem maintenance (GO:0010073); Biological Process: leaf vascular tissue pattern formation (GO:0010305); Biological Process: basipetal auxin transport (GO:0010540); Biological Process: plant-type cell wall cellulose metabolic process (GO:0052541); Biological Process: cell wall pectin metabolic process (GO:0052546); | ######## | 0.999991 |
| 26 | BnaA03g48640D | -- | Molecular Function: GTPase activity (GO:0003924); Molecular Function: GTP binding (GO:0005525); Cellular Component: endoplasmic reticulum membrane (GO:0005789); Cellular Component: cytosol (GO:0005829); Cellular Component: plasma membrane (GO:0005886); Biological Process: protein targeting to membrane (GO:0006612); Biological Process: nucleotide transport (GO:0006862); Biological Process: ER to Golgi vesicle-mediated transport (GO:0006888); Biological Process: membrane fusion (GO:0006944); Cellular Component: plasmodesma (GO:0009506); Cellular Component: chloroplast outer membrane (GO:0009707); Biological Process: regulation of proton transport (GO:0010155); Biological Process: regulation of plant-type hypersensitive response (GO:0010363); Biological Process: ammonium transport (GO:0015696); Biological Process: basic amino acid transport (GO:0015802); Molecular Function: protein homodimerization activity (GO:0042803); Biological Process: negative regulation of programmed cell death (GO:0043069); Biological Process: amino acid import (GO:0043090); Biological Process: protein targeting to chloroplast (GO:0045036); | 0.035989 | 1.025259 |
| 27 | BnaA03g52100D | -- | Molecular Function: inorganic anion exchanger activity (GO:0005452); Cellular Component: nucleus (GO:0005634); Cellular Component: mitochondrion (GO:0005739); Cellular Component: integral component of membrane (GO:0016021); Biological Process: borate transmembrane transport (GO:0035445); Molecular Function: borate transmembrane transporter activity (GO:0046715); | -0.18724 | 0.878285 |
| 28 | BnaA03g52970D | K02984\|4.3109e-178\|brp:103862366\|40S ribosomal protein S3a-2-like; K02984 small subunit ribosomal protein S3Ae (A) | Biological Process: RNA methylation (GO:0001510); Molecular Function: structural constituent of ribosome (GO:0003735); Cellular Component: cell wall (GO:0005618); Cellular Component: nucleolus (GO:0005730); Cellular Component: Golgi apparatus (GO:0005794); Cellular Component: plasma membrane (GO:0005886); Biological Process: translation (GO:0006412); Cellular Component: plasmodesma (GO:0009506); Cellular Component: cytosolic small ribosomal subunit (GO:0022627); | -0.10219 | 0.931616 |
| 29 | BnaA03g55010D | -- | Biological Process: abscisic acid biosynthetic process (GO:0009688); | 0.042386 | 1.029816 |
| 30 | BnaA04g10940D | K04730\|2.50783e-17\|aly:ARALYDRAFT_667762\|hypothetical protein; K04730 interleukin-1 receptor-associated kinase 1 [EC:2.7.11.1] (A) | -- | -- | -- |
| 31 | BnaA04g26090D | K00800\|0\|brp:103866199\|3-phosphoshikimate 1-carboxyvinyltransferase, chloroplastic; K00800 3-phosphoshikimate 1-carboxyvinyltransferase [EC:2.5.1.19] (A) | Molecular Function: 3-phosphoshikimate 1-carboxyvinyltransferase activity (GO:0003866); Cellular Component: cytosol (GO:0005829); Biological Process: aromatic amino acid family biosynthetic process (GO:0009073); Biological Process: chorismate biosynthetic process (GO:0009423); Cellular Component: chloroplast stroma (GO:0009570); Biological Process: glyphosate metabolic process (GO:0018920); | -0.06787 | 0.954042 |
| 32 | BnaA04g27000D | K11797\|0\|brp:103843746\|PH-interacting protein; K11797 PH-interacting protein (A) | Molecular Function: nucleotide binding (GO:0000166); Cellular Component: nucleus (GO:0005634); Biological Process: gravitropism (GO:0009630); Cellular Component: Cul4-RING E3 ubiquitin ligase complex (GO:0080008); | 0.0146 | 1.010171 |
| 33 | BnaA04g27680D | -- | Biological Process: signal transduction (GO:0007165); Molecular Function: phosphatidylinositol binding (GO:0035091); | 0.090984 | 1.065096 |
| 34 | BnaA04g29130D | K11808\|0\|brp:103865624\|phosphoribosylaminoimidazole carboxylase, chloroplastic; K11808 phosphoribosylaminoimidazole carboxylase [EC:4.1.1.21] (A) | Molecular Function: phosphoribosylaminoimidazole carboxylase activity (GO:0004638); Molecular Function: ATP binding (GO:0005524); Cellular Component: mitochondrion (GO:0005739); Biological Process: 'de novo' IMP biosynthetic process (GO:0006189); Biological Process: pollen development (GO:0009555); Cellular Component: chloroplast stroma (GO:0009570); Molecular Function: 5-(carboxyamino)imidazole ribonucleotide mutase activity (GO:0034023); Molecular Function: metal ion binding (GO:0046872); | -0.07331 | 0.950452 |
| 35 | BnaA05g00920D | -- | Molecular Function: sequence-specific DNA binding transcription factor activity (GO:0003700); Cellular Component: nucleus (GO:0005634); Biological Process: regulation of transcription, DNA-templated (GO:0006355); Biological Process: shade avoidance (GO:0009641); Biological Process: red or far-red light signaling pathway (GO:0010017); Molecular Function: protein dimerization activity (GO:0046983); | -0.57384 | 0.671828 |
| 36 | BnaA05g05630D | K12947\|6.25797e-129\|brp:103866994\|probable signal peptidase complex subunit 2; K12947 signal peptidase complex subunit 2 [EC:3.4.-.-] (A) | Cellular Component: nucleus (GO:0005634); Cellular Component: signal peptidase complex (GO:0005787); Biological Process: signal peptide processing (GO:0006465); Molecular Function: peptidase activity (GO:0008233); Cellular Component: integral component of membrane (GO:0016021); Biological Process: second-messenger-mediated signaling (GO:0019932); | -0.02279 | 0.984327 |
| 37 | BnaA05g10740D | K00902\|0\|brp:103867627\|dolichol kinase-like; K00902 dolichol kinase [EC:2.7.1.108] (A) | Molecular Function: dolichol kinase activity (GO:0004168); Molecular Function: phosphatidate cytidylyltransferase activity (GO:0004605); Biological Process: membrane fusion (GO:0006944); Biological Process: phospholipid biosynthetic process (GO:0008654); Biological Process: salicylic acid biosynthetic process (GO:0009697); Biological Process: salicylic acid mediated signaling pathway (GO:0009863); Biological Process: response to chitin (GO:0010200); Cellular Component: membrane (GO:0016020); Biological Process: endoplasmic reticulum unfolded protein response (GO:0030968); Biological Process: negative regulation of defense response (GO:0031348); Biological Process: innate immune response (GO:0045087); | -0.0291 | 0.980032 |
| 38 | BnaA05g14600D | -- | Cellular Component: plant-type vacuole (GO:0000325); Molecular Function: calcium:sodium antiporter activity (GO:0005432); Molecular Function: calcium ion binding (GO:0005509); Cellular Component: vacuolar membrane (GO:0005774); Biological Process: Golgi organization (GO:0007030); Biological Process: vacuole organization (GO:0007033); Cellular Component: plasmodesma (GO:0009506); Cellular Component: chloroplast (GO:0009507); Cellular Component: integral component of membrane (GO:0016021); Biological Process: hydrogen peroxide catabolic process (GO:0042744); Biological Process: calcium ion homeostasis (GO:0055074); Biological Process: transmembrane transport (GO:0055085); Biological Process: cellular response to salt stress (GO:0071472); | -0.00606 | 0.995811 |
| 39 | BnaA05g26430D | -- | Cellular Component: Golgi apparatus (GO:0005794); Biological Process: response to ethylene (GO:0009723); Biological Process: abscisic acid-activated signaling pathway (GO:0009738); Biological Process: phospholipid transport (GO:0015914); Cellular Component: membrane (GO:0016020); Biological Process: intracellular signal transduction (GO:0035556); | 0.286314 | 1.21952 |
| 40 | BnaA05g30800D | K18674\|0\|brp:103870735\|galactokinase; K18674 N-acetylgalactosamine kinase [EC:2.7.1.157] (A) | Molecular Function: galactokinase activity (GO:0004335); Molecular Function: ATP binding (GO:0005524); Cellular Component: cytosol (GO:0005829); Biological Process: galactose metabolic process (GO:0006012); Molecular Function: N-acetylgalactosamine kinase activity (GO:0033858); Biological Process: carbohydrate phosphorylation (GO:0046835); | 0.150464 | 1.109926 |
| 41 | BnaA06g00730D | -- | Cellular Component: mitochondrion (GO:0005739); Cellular Component: vacuolar membrane (GO:0005774); Cellular Component: plastid (GO:0009536); | -0.05997 | 0.959283 |
| 42 | BnaA06g02140D | K00262\|0\|brp:103871264\|NADP-specific glutamate dehydrogenase; K00262 glutamate dehydrogenase (NADP+) [EC:1.4.1.4] (A) | Molecular Function: nucleotide binding (GO:0000166); Molecular Function: glutamate dehydrogenase (NADP+) activity (GO:0004354); Cellular Component: cytoplasm (GO:0005737); Biological Process: cellular amino acid metabolic process (GO:0006520); Biological Process: spermidine biosynthetic process (GO:0008295); Biological Process: glucuronoxylan metabolic process (GO:0010413); Biological Process: xylan biosynthetic process (GO:0045492); Biological Process: oxidation-reduction process (GO:0055114); | -0.22486 | 0.855676 |
| 43 | BnaA06g03600D | K14442\|0\|brp:103871455\|ATP-dependent RNA helicase DHX36; K14442 ATP-dependent RNA helicase DHX36 [EC:3.6.4.13] (A) | Molecular Function: double-stranded RNA binding (GO:0003725); Molecular Function: ATP binding (GO:0005524); Cellular Component: intracellular (GO:0005622); Molecular Function: ATP-dependent helicase activity (GO:0008026); | -0.06683 | 0.95473 |
| 44 | BnaA06g13080D | K13412\|0\|brp:103872695\|calcium-dependent protein kinase 10-like; K13412 calcium-dependent protein kinase [EC:2.7.11.1] (A) | Biological Process: MAPK cascade (GO:0000165); Molecular Function: calmodulin-dependent protein kinase activity (GO:0004683); Molecular Function: calcium ion binding (GO:0005509); Molecular Function: ATP binding (GO:0005524); Cellular Component: nucleus (GO:0005634); Biological Process: N-terminal protein myristoylation (GO:0006499); Biological Process: protein targeting to membrane (GO:0006612); Biological Process: membrane fusion (GO:0006944); Biological Process: response to cold (GO:0009409); Biological Process: response to water deprivation (GO:0009414); Biological Process: response to wounding (GO:0009611); Biological Process: salicylic acid biosynthetic process (GO:0009697); Biological Process: response to ethylene (GO:0009723); Biological Process: response to auxin (GO:0009733); Biological Process: abscisic acid-activated signaling pathway (GO:0009738); Biological Process: systemic acquired resistance, salicylic acid mediated signaling pathway (GO:0009862); Biological Process: jasmonic acid mediated signaling pathway (GO:0009867); Biological Process: regulation of plant-type hypersensitive response (GO:0010363); Biological Process: endoplasmic reticulum unfolded protein response (GO:0030968); Biological Process: negative regulation of defense response (GO:0031348); Biological Process: hyperosmotic salinity response (GO:0042538); Biological Process: negative regulation of programmed cell death (GO:0043069); Biological Process: protein autophosphorylation (GO:0046777); Biological Process: defense response to fungus (GO:0050832); | -0.03668 | 0.974898 |
| 45 | BnaA06g22840D | -- | Molecular Function: G-protein coupled receptor kinase activity (GO:0004703); Molecular Function: ATP binding (GO:0005524); Cellular Component: plasma membrane (GO:0005886); Biological Process: protein phosphorylation (GO:0006468); Biological Process: protein targeting to membrane (GO:0006612); Biological Process: transmembrane receptor protein tyrosine kinase signaling pathway (GO:0007169); Biological Process: positive regulation of flavonoid biosynthetic process (GO:0009963); Biological Process: regulation of plant-type hypersensitive response (GO:0010363); Cellular Component: integral component of membrane (GO:0016021); | -0.0674 | 0.954357 |
| 46 | BnaA06g24600D | K01610\|0\|brp:103874006\|phosphoenolpyruvate carboxykinase [ATP]; K01610 phosphoenolpyruvate carboxykinase (ATP) [EC:4.1.1.49] (A) | Molecular Function: phosphoenolpyruvate carboxykinase (ATP) activity (GO:0004612); Molecular Function: ATP binding (GO:0005524); Cellular Component: cytoplasm (GO:0005737); Biological Process: gluconeogenesis (GO:0006094); Molecular Function: kinase activity (GO:0016301); Biological Process: phosphorylation (GO:0016310); | -- | -- |
| 47 | BnaA06g25730D | K05868\|0\|brp:103874112\|cyclin-B2-1-like; K05868 cyclin B (A) | Biological Process: regulation of cyclin-dependent protein serine/threonine kinase activity (GO:0000079); Cellular Component: nucleus (GO:0005634); Biological Process: DNA replication (GO:0006260); Biological Process: DNA methylation (GO:0006306); Biological Process: cell proliferation (GO:0008283); Biological Process: stomatal lineage progression (GO:0010440); Molecular Function: cyclin-dependent protein serine/threonine kinase regulator activity (GO:0016538); Molecular Function: protein kinase binding (GO:0019901); Biological Process: cell division (GO:0051301); Biological Process: histone H3-K9 methylation (GO:0051567); | -0.25207 | 0.839691 |
| 48 | BnaA06g27490D | K00517\|0\|eus:EUTSA_v10004073mg\|hypothetical protein; K00517 [EC:1.14.-.-] (A) | Molecular Function: binding (GO:0005488); Cellular Component: plasma membrane (GO:0005886); Molecular Function: oxidoreductase activity (GO:0016491); | -- | -- |
| 49 | BnaA06g33370D | K03456\|0\|brp:103875349\|serine/threonine-protein phosphatase 2A 65 kDa regulatory subunit A beta isoform; K03456 serine/threonine-protein phosphatase 2A regulatory subunit A (A) | Cellular Component: protein phosphatase type 2A complex (GO:0000159); Biological Process: cell morphogenesis (GO:0000902); Cellular Component: cell wall (GO:0005618); Cellular Component: cytosol (GO:0005829); Cellular Component: plasma membrane (GO:0005886); Biological Process: N-terminal protein myristoylation (GO:0006499); Molecular Function: protein phosphatase type 2A regulator activity (GO:0008601); Biological Process: cell growth (GO:0016049); Biological Process: regulation of phosphorylation (GO:0042325); Biological Process: response to cadmium ion (GO:0046686); Biological Process: Golgi vesicle transport (GO:0048193); | 0.133791 | 1.097173 |
| 50 | BnaA06g35490D | -- | Molecular Function: transporter activity (GO:0005215); Cellular Component: intracellular (GO:0005622); Cellular Component: plasma membrane (GO:0005886); Biological Process: transport (GO:0006810); | 0.617732 | 1.534461 |
| 51 | BnaA06g36030D | -- | Cellular Component: mitochondrion (GO:0005739); Cellular Component: Golgi apparatus (GO:0005794); Biological Process: plant-type secondary cell wall biogenesis (GO:0009834); Biological Process: glucuronoxylan metabolic process (GO:0010413); Biological Process: xylan biosynthetic process (GO:0045492); | 0.124707 | 1.090286 |
| 52 | BnaA06g36090D | -- | Molecular Function: acetyl-CoA C-acyltransferase activity (GO:0003988); Molecular Function: inward rectifier potassium channel activity (GO:0005242); Molecular Function: protein binding (GO:0005515); Cellular Component: nucleus (GO:0005634); Cellular Component: mitochondrion (GO:0005739); Cellular Component: vacuole (GO:0005773); Cellular Component: peroxisome (GO:0005777); Cellular Component: plasma membrane (GO:0005886); Biological Process: potassium ion transport (GO:0006813); Cellular Component: integral component of membrane (GO:0016021); Molecular Function: cyclic nucleotide binding (GO:0030551); Biological Process: transmembrane transport (GO:0055085); | -0.06244 | 0.957645 |
| 53 | BnaA06g36270D | -- | Molecular Function: sequence-specific DNA binding transcription factor activity (GO:0003700); Molecular Function: protein binding (GO:0005515); Cellular Component: nucleus (GO:0005634); Biological Process: regulation of transcription, DNA-templated (GO:0006355); Biological Process: gravitropism (GO:0009630); Biological Process: vegetative to reproductive phase transition of meristem (GO:0010228); Molecular Function: sequence-specific DNA binding (GO:0043565); | 0.111669 | 1.080478 |
| 54 | BnaA06g37410D | -- | Molecular Function: beta-galactosidase activity (GO:0004565); Cellular Component: extracellular region (GO:0005576); Biological Process: carbohydrate metabolic process (GO:0005975); Biological Process: plant-type cell wall modification (GO:0009827); Biological Process: pollen tube growth (GO:0009860); Molecular Function: carbohydrate binding (GO:0030246); Molecular Function: cation binding (GO:0043169); | -0.33644 | 0.791993 |
| 55 | BnaA07g06530D | -- | Cellular Component: Golgi apparatus (GO:0005794); Cellular Component: trans-Golgi network (GO:0005802); Cellular Component: plasma membrane (GO:0005886); Biological Process: phosphate ion transport (GO:0006817); Biological Process: iron ion transport (GO:0006826); Biological Process: response to wounding (GO:0009611); Biological Process: coumarin biosynthetic process (GO:0009805); Biological Process: cellular response to iron ion starvation (GO:0010106); Biological Process: response to nitrate (GO:0010167); Molecular Function: phosphate ion transmembrane transporter activity (GO:0015114); Biological Process: nitrate transport (GO:0015706); Cellular Component: integral component of membrane (GO:0016021); Biological Process: cellular response to phosphate starvation (GO:0016036); | 0.234805 | 1.176747 |
| 56 | BnaA07g16890D | -- | Cellular Component: nucleus (GO:0005634); Cellular Component: plasma membrane (GO:0005886); Biological Process: circadian rhythm (GO:0007623); | 0.191662 | 1.142078 |
| 57 | BnaA07g24730D | -- | Cellular Component: nucleus (GO:0005634); | -0.00515 | 0.996436 |
| 58 | BnaA07g34460D | -- | Cellular Component: plasma membrane (GO:0005886); | -- | -- |
| 59 | BnaA07g35190D | K13100\|0\|brp:103832444\|pre-mRNA-splicing factor CWC22 homolog; K13100 pre-mRNA-splicing factor CWC22 (A) | Molecular Function: DNA binding (GO:0003677); Molecular Function: RNA binding (GO:0003723); Cellular Component: nucleus (GO:0005634); Cellular Component: cytosol (GO:0005829); Biological Process: translation (GO:0006412); Biological Process: RNA metabolic process (GO:0016070); | 0.162722 | 1.119397 |
| 60 | BnaA07g36460D | -- | Cellular Component: vacuole (GO:0005773); Cellular Component: chloroplast (GO:0009507); | -0.08966 | 0.939742 |
| 61 | BnaA07g38460D | -- | Cellular Component: nucleus (GO:0005634); Biological Process: sterol biosynthetic process (GO:0016126); Biological Process: pentacyclic triterpenoid biosynthetic process (GO:0019745); Molecular Function: lupeol synthase activity (GO:0042299); Molecular Function: beta-amyrin synthase activity (GO:0042300); | -0.08082 | 0.945518 |
| 62 | BnaA08g14610D | K03037\|0\|brp:103834895\|26S proteasome non-ATPase regulatory subunit 6 homolog; K03037 26S proteasome regulatory subunit N7 (A) | Biological Process: mitotic G2 phase (GO:0000085); Cellular Component: nucleus (GO:0005634); Cellular Component: cytosol (GO:0005829); Cellular Component: plasma membrane (GO:0005886); Biological Process: fatty acid beta-oxidation (GO:0006635); Biological Process: ER to Golgi vesicle-mediated transport (GO:0006888); Cellular Component: proteasome regulatory particle, lid subcomplex (GO:0008541); Biological Process: toxin catabolic process (GO:0009407); Cellular Component: chloroplast (GO:0009507); Biological Process: photomorphogenesis (GO:0009640); Biological Process: response to cytokinin (GO:0009735); Biological Process: trichome morphogenesis (GO:0010090); Biological Process: cullin deneddylation (GO:0010388); Biological Process: DNA endoreduplication (GO:0042023); Biological Process: amino acid import (GO:0043090); Biological Process: proteasome-mediated ubiquitin-dependent protein catabolic process (GO:0043161); Biological Process: root hair elongation (GO:0048767); Biological Process: regulation of cell division (GO:0051302); Biological Process: regulation of unidimensional cell growth (GO:0051510); Biological Process: protein maturation (GO:0051604); Biological Process: response to misfolded protein (GO:0051788); Biological Process: proteasome core complex assembly (GO:0080129); | -0.05677 | 0.961411 |
| 63 | BnaA08g21550D | -- | Molecular Function: DNA binding (GO:0003677); Molecular Function: sequence-specific DNA binding transcription factor activity (GO:0003700); Cellular Component: nucleus (GO:0005634); Biological Process: embryo sac egg cell differentiation (GO:0009560); Biological Process: post-translational protein modification (GO:0043687); Biological Process: positive regulation of transcription, DNA-templated (GO:0045893); | -0.1821 | 0.88142 |
| 64 | BnaA08g22900D | -- | -- | 0.112533 | 1.081125 |
| 65 | BnaA08g23890D | -- | Cellular Component: nucleus (GO:0005634); Cellular Component: chloroplast (GO:0009507); | -0.01724 | 0.988122 |
| 66 | BnaA08g27230D | -- | Molecular Function: polygalacturonase activity (GO:0004650); Cellular Component: extracellular region (GO:0005576); Biological Process: carbohydrate metabolic process (GO:0005975); | -0.23348 | 0.85058 |
| 67 | BnaA08g28700D | -- | Cellular Component: nucleus (GO:0005634); Biological Process: regulation of transcription, DNA-templated (GO:0006355); Biological Process: male meiosis II (GO:0007142); Biological Process: microsporogenesis (GO:0009556); | 0.933071 | 1.909336 |
| 68 | BnaA08g30200D | K11314\|0\|brp:103849520\|transcriptional adapter ADA2b; K11314 transcriptional adapter 2-alpha (A) | Biological Process: mitotic cell cycle (GO:0000278); Biological Process: nuclear-transcribed mRNA catabolic process (GO:0000956); Molecular Function: DNA binding (GO:0003677); Molecular Function: chromatin binding (GO:0003682); Molecular Function: sequence-specific DNA binding transcription factor activity (GO:0003700); Molecular Function: transcription coactivator activity (GO:0003713); Molecular Function: protein binding (GO:0005515); Cellular Component: nucleus (GO:0005634); Biological Process: methylation-dependent chromatin silencing (GO:0006346); Biological Process: RNA processing (GO:0006396); Molecular Function: zinc ion binding (GO:0008270); Biological Process: positive regulation of cell proliferation (GO:0008284); Biological Process: cold acclimation (GO:0009631); Biological Process: response to high light intensity (GO:0009644); Biological Process: response to auxin (GO:0009733); Biological Process: response to cytokinin (GO:0009735); Biological Process: RNA interference (GO:0016246); Biological Process: response to hydrogen peroxide (GO:0042542); | -0.01832 | 0.987379 |
| 69 | BnaA08g30570D | -- | Molecular Function: translation initiation factor activity (GO:0003743); Biological Process: rRNA processing (GO:0006364); Biological Process: translational initiation (GO:0006413); Cellular Component: chloroplast thylakoid (GO:0009534); Biological Process: response to blue light (GO:0009637); Biological Process: response to red light (GO:0010114); Biological Process: photosystem II assembly (GO:0010207); Biological Process: response to far red light (GO:0010218); Biological Process: carotenoid biosynthetic process (GO:0016117); Biological Process: isopentenyl diphosphate biosynthetic process, methylerythritol 4-phosphate pathway (GO:0019288); | 0.006847 | 1.004757 |
| 70 | BnaA08g31640D | K03327\|0\|brp:103836219\|protein TRANSPARENT TESTA 12-like; K03327 multidrug resistance protein, MATE family (A) | Cellular Component: plasma membrane (GO:0005886); Biological Process: drug transmembrane transport (GO:0006855); Cellular Component: chloroplast (GO:0009507); Biological Process: response to salt stress (GO:0009651); Biological Process: jasmonic acid metabolic process (GO:0009694); Biological Process: response to abscisic acid (GO:0009737); Biological Process: response to jasmonic acid (GO:0009753); Biological Process: response to chitin (GO:0010200); Molecular Function: drug transmembrane transporter activity (GO:0015238); Molecular Function: antiporter activity (GO:0015297); Biological Process: proline transport (GO:0015824); Biological Process: response to karrikin (GO:0080167); | -- | -- |
| 71 | BnaA09g03590D | K14565\|0\|brp:103837322\|probable nucleolar protein 5-1; K14565 nucleolar protein 58 (A) | Biological Process: RNA methylation (GO:0001510); Molecular Function: DNA binding (GO:0003677); Cellular Component: nucleolus (GO:0005730); Cellular Component: cytosol (GO:0005829); Biological Process: purine nucleotide biosynthetic process (GO:0006164); Biological Process: pyrimidine ribonucleotide biosynthetic process (GO:0009220); Cellular Component: plasmodesma (GO:0009506); Cellular Component: membrane (GO:0016020); | -0.03564 | 0.975602 |
| 72 | BnaA09g05820D | -- | Cellular Component: nucleus (GO:0005634); | 1.090279 | 2.129152 |
| 73 | BnaA09g08460D | -- | Biological Process: mitotic nuclear division (GO:0007067); Molecular Function: zinc ion binding (GO:0008270); Biological Process: response to abscisic acid (GO:0009737); | 0.002589 | 1.001796 |
| 74 | BnaA09g10030D | -- | Biological Process: regulation of cell growth by extracellular stimulus (GO:0001560); Cellular Component: cytoplasm (GO:0005737); Biological Process: pentose-phosphate shunt (GO:0006098); Biological Process: glycine catabolic process (GO:0006546); Biological Process: tryptophan catabolic process (GO:0006569); Biological Process: unsaturated fatty acid biosynthetic process (GO:0006636); Biological Process: vitamin metabolic process (GO:0006766); Molecular Function: transaminase activity (GO:0008483); Biological Process: lipoate metabolic process (GO:0009106); Biological Process: coenzyme biosynthetic process (GO:0009108); Biological Process: indoleacetic acid biosynthetic process (GO:0009684); Biological Process: jasmonic acid biosynthetic process (GO:0009695); Cellular Component: membrane (GO:0016020); Molecular Function: 1-aminocyclopropane-1-carboxylate synthase activity (GO:0016847); Biological Process: starch biosynthetic process (GO:0019252); Biological Process: isopentenyl diphosphate biosynthetic process, methylerythritol 4-phosphate pathway (GO:0019288); Biological Process: cysteine biosynthetic process (GO:0019344); Biological Process: glucosinolate biosynthetic process (GO:0019761); Molecular Function: pyridoxal phosphate binding (GO:0030170); Biological Process: 1-aminocyclopropane-1-carboxylate biosynthetic process (GO:0042218); Biological Process: defense response to bacterium (GO:0042742); Biological Process: adventitious root development (GO:0048830); Molecular Function: S-alkylthiohydroximate lyase activity (GO:0080108); | 0.496224 | 1.410517 |
| 75 | BnaA09g15460D | -- | Molecular Function: nucleic acid binding (GO:0003676); Cellular Component: nucleus (GO:0005634); Biological Process: nucleobase-containing compound metabolic process (GO:0006139); Molecular Function: 3'-5' exonuclease activity (GO:0008408); | 0.264628 | 1.201326 |
| 76 | BnaA09g18270D | -- | Cellular Component: nucleus (GO:0005634); | 0.341441 | 1.267021 |
| 77 | BnaA09g19540D | K12891\|1.74459e-06\|crb:CARUB_v10026844mg\|hypothetical protein; K12891 splicing factor, arginine/serine-rich 2 (A) | -- | -0.16707 | 0.89065 |
| 78 | BnaA09g20670D | K12483\|0\|brp:103839856\|EH domain-containing protein 1; K12483 EH domain-containing protein 1 (A) | Molecular Function: GTPase activity (GO:0003924); Molecular Function: calcium ion binding (GO:0005509); Molecular Function: GTP binding (GO:0005525); Cellular Component: cytoplasm (GO:0005737); Cellular Component: plasma membrane (GO:0005886); Biological Process: GTP catabolic process (GO:0006184); Biological Process: endocytosis (GO:0006897); Cellular Component: plasmodesma (GO:0009506); Cellular Component: intracellular membrane-bounded organelle (GO:0043231); | 0.144936 | 1.105682 |
| 79 | BnaA09g22640D | -- | Molecular Function: serine-type endopeptidase activity (GO:0004252); Cellular Component: extracellular region (GO:0005576); Cellular Component: cell wall (GO:0005618); Biological Process: proteolysis (GO:0006508); Cellular Component: chloroplast (GO:0009507); Molecular Function: identical protein binding (GO:0042802); Biological Process: negative regulation of catalytic activity (GO:0043086); | -- | -- |
| 80 | BnaA09g24450D | K08488\|2.13987e-148\|brp:103840170\|syntaxin-22; K08488 syntaxin 7 (A) | Molecular Function: SNAP receptor activity (GO:0005484); Biological Process: intracellular protein transport (GO:0006886); Cellular Component: membrane (GO:0016020); Biological Process: vesicle-mediated transport (GO:0016192); | 0.102316 | 1.073495 |
| 81 | BnaA09g26290D | K11593\|8.53762e-09\|brp:103845605\|protein argonaute 9-like; K11593 eukaryotic translation initiation factor 2C (A) | -- | 0.128722 | 1.093325 |
| 82 | BnaA09g30400D | -- | Cellular Component: extracellular region (GO:0005576); Cellular Component: mitochondrion (GO:0005739); Cellular Component: vacuole (GO:0005773); | 0.198321 | 1.147362 |
| 83 | BnaA09g31630D | -- | Biological Process: nuclear division (GO:0000280); Biological Process: cytokinesis by cell plate formation (GO:0000911); Cellular Component: nucleus (GO:0005634); Cellular Component: cytoplasm (GO:0005737); Biological Process: chromatin silencing (GO:0006342); Biological Process: nucleolus organization (GO:0007000); Biological Process: histone phosphorylation (GO:0016572); Biological Process: cortical microtubule organization (GO:0043622); Biological Process: cell redox homeostasis (GO:0045454); Molecular Function: beta-tubulin binding (GO:0048487); Biological Process: anisotropic cell growth (GO:0051211); Biological Process: histone H3-K9 methylation (GO:0051567); | -0.00578 | 0.996002 |
| 84 | BnaA09g35940D | K14785\|4.37098e-175\|brp:103841555\|pre-rRNA-processing protein ESF2; K14785 ESF2/ABP1 family protein (A) | Molecular Function: nucleotide binding (GO:0000166); Molecular Function: nucleic acid binding (GO:0003676); Cellular Component: nucleus (GO:0005634); Molecular Function: TBP-class protein binding (GO:0017025); | 0.320935 | 1.24914 |
| 85 | BnaA09g42440D | K13162\|0\|brp:103837876\|KH domain-containing protein At4g18375-like; K13162 poly(rC)-binding protein 2/3/4 (A) | Molecular Function: RNA binding (GO:0003723); Cellular Component: nucleus (GO:0005634); | -0.24618 | 0.843127 |
| 86 | BnaA09g44720D | K03267\|0\|brp:103842720\|eukaryotic peptide chain release factor GTP-binding subunit ERF3A-like; K03267 peptide chain release factor subunit 3 (A) | Molecular Function: translation release factor activity (GO:0003747); Molecular Function: GTPase activity (GO:0003924); Molecular Function: GTP binding (GO:0005525); Cellular Component: vacuole (GO:0005773); Cellular Component: cytosol (GO:0005829); Biological Process: GTP catabolic process (GO:0006184); Biological Process: translational termination (GO:0006415); | -0.32647 | 0.797484 |
| 87 | BnaA09g45520D | -- | Cellular Component: cytosol (GO:0005829); | 0.024893 | 1.017404 |
| 88 | BnaA09g46120D | -- | Molecular Function: acid phosphatase activity (GO:0003993); Molecular Function: protein serine/threonine phosphatase activity (GO:0004722); Cellular Component: extracellular region (GO:0005576); Biological Process: tryptophan catabolic process (GO:0006569); Biological Process: systemic acquired resistance (GO:0009627); Biological Process: indoleacetic acid biosynthetic process (GO:0009684); Biological Process: regulation of defense response (GO:0031347); Molecular Function: metal ion binding (GO:0046872); | 0.408291 | 1.327113 |
| 89 | BnaA09g48540D | -- | -- | -2.31215 | 0.20136 |
| 90 | BnaA09g49060D | -- | -- | 0.117531 | 1.084876 |
| 91 | BnaA09g53140D | -- | Molecular Function: N,N-dimethylaniline monooxygenase activity (GO:0004499); Cellular Component: nucleus (GO:0005634); Cellular Component: vacuole (GO:0005773); Molecular Function: flavin adenine dinucleotide binding (GO:0050660); Molecular Function: NADP binding (GO:0050661); Biological Process: oxidation-reduction process (GO:0055114); | 0.069428 | 1.049301 |
| 92 | BnaA10g11050D | K06444\|0\|brp:103845139\|lycopene epsilon cyclase, chloroplastic-like; K06444 lycopene epsilon-cyclase [EC:5.5.1.18] (A) | Cellular Component: chloroplast (GO:0009507); Biological Process: photosynthesis, light harvesting (GO:0009765); Biological Process: stomatal complex morphogenesis (GO:0010103); Biological Process: carotene biosynthetic process (GO:0016120); Biological Process: xanthophyll biosynthetic process (GO:0016123); Molecular Function: oxidoreductase activity, acting on paired donors, with incorporation or reduction of molecular oxygen (GO:0016705); Molecular Function: lycopene epsilon cyclase activity (GO:0045435); | 0.273017 | 1.208332 |
| 93 | BnaA10g12000D | -- | Cellular Component: extracellular region (GO:0005576); Cellular Component: nucleus (GO:0005634); Cellular Component: vacuolar membrane (GO:0005774); | -0.05995 | 0.959294 |
| 94 | BnaA10g16410D | K17601\|0\|brp:103845826\|probable inactive serine/threonine-protein kinase lvsG; K17601 WD repeat-containing protein 81 (A) | Cellular Component: nucleus (GO:0005634); Biological Process: protein N-linked glycosylation (GO:0006487); Cellular Component: Cul4-RING E3 ubiquitin ligase complex (GO:0080008); | 0.128846 | 1.093419 |
| 95 | BnaA10g26570D | -- | Cellular Component: cytoplasm (GO:0005737); Biological Process: Mo-molybdopterin cofactor biosynthetic process (GO:0006777); Molecular Function: transferase activity (GO:0016740); Biological Process: glucosinolate biosynthetic process (GO:0019761); | 0.052781 | 1.037262 |
| 96 | BnaA10g27110D | K03857\|0\|brp:103847450\|phosphatidylinositol N-acetylglucosaminyltransferase gpi3 subunit; K03857 phosphatidylinositol glycan, class A [EC:2.4.1.198] (A) | Cellular Component: nucleus (GO:0005634); Biological Process: GPI anchor biosynthetic process (GO:0006506); Molecular Function: transferase activity, transferring glycosyl groups (GO:0016757); | -0.21873 | 0.859319 |
| 97 | BnaA10g27470D | -- | Cellular Component: Golgi apparatus (GO:0005794); Molecular Function: transferase activity, transferring glycosyl groups (GO:0016757); | -- | -- |
| 98 | BnaAnng03050D | K11647\|0\|brp:103849994\|probable ATP-dependent DNA helicase CHR12; K11647 SWI/SNF-related matrix-associated actin-dependent regulator of chromatin subfamily A member 2/4 [EC:3.6.4.-] (A) | Biological Process: mitotic G2 phase (GO:0000085); Biological Process: nuclear-transcribed mRNA catabolic process (GO:0000956); Molecular Function: DNA binding (GO:0003677); Molecular Function: helicase activity (GO:0004386); Molecular Function: ATP binding (GO:0005524); Cellular Component: nucleus (GO:0005634); Biological Process: DNA metabolic process (GO:0006259); Biological Process: RNA processing (GO:0006396); Biological Process: sister chromatid cohesion (GO:0007062); Biological Process: response to heat (GO:0009408); Biological Process: response to water deprivation (GO:0009414); Biological Process: photomorphogenesis (GO:0009640); Biological Process: response to salt stress (GO:0009651); Biological Process: cullin deneddylation (GO:0010388); Biological Process: protein ubiquitination (GO:0016567); Biological Process: histone methylation (GO:0016571); Biological Process: protein deubiquitination (GO:0016579); Biological Process: chromatin silencing by small RNA (GO:0031048); Biological Process: regulation of chromosome organization (GO:0033044); Biological Process: meiotic chromosome segregation (GO:0045132); Biological Process: positive regulation of transcription, DNA-templated (GO:0045893); | 0.115016 | 1.082987 |
| 99 | BnaAnng13790D | -- | -- | 5.034997 | 32.78574 |
| 100 | BnaAnng16670D | -- | Molecular Function: hydrolase activity, hydrolyzing O-glycosyl compounds (GO:0004553); Cellular Component: extracellular region (GO:0005576); Cellular Component: Golgi apparatus (GO:0005794); Cellular Component: cytosol (GO:0005829); Cellular Component: plasma membrane (GO:0005886); Cellular Component: plant-type cell wall (GO:0009505); Cellular Component: plasmodesma (GO:0009506); | -0.09178 | 0.938365 |
| 101 | BnaAnng18890D | K13201\|0\|brp:103842756\|oligouridylate-binding protein 1B; K13201 nucleolysin TIA-1/TIAR (A) | Molecular Function: nucleotide binding (GO:0000166); Molecular Function: mRNA 3'-UTR binding (GO:0003730); Cellular Component: cytosol (GO:0005829); | -0.7672 | 0.587557 |
| 102 | BnaAnng23210D | -- | Cellular Component: Golgi apparatus (GO:0005794); Molecular Function: transferase activity, transferring glycosyl groups (GO:0016757); | 0.069342 | 1.049238 |
| 103 | BnaAnng30390D | K00852\|0\|brp:103872503\|putative ribokinase; K00852 ribokinase [EC:2.7.1.15] (A) | Molecular Function: ribokinase activity (GO:0004747); Biological Process: D-ribose metabolic process (GO:0006014); Cellular Component: chloroplast stroma (GO:0009570); Biological Process: phosphorylation (GO:0016310); | -0.08238 | 0.944499 |
| 104 | BnaAnng31300D | -- | -- | 0.059777 | 1.042304 |
| 105 | BnaAnng39330D | -- | Molecular Function: protein serine/threonine kinase activity (GO:0004674); Molecular Function: ATP binding (GO:0005524); Cellular Component: nucleus (GO:0005634); Cellular Component: plasma membrane (GO:0005886); Biological Process: protein phosphorylation (GO:0006468); Biological Process: auxin-activated signaling pathway (GO:0009734); | -0.39345 | 0.761305 |
| 106 | BnaAnng40810D | K07466\|9.15026e-27\|brp:103841154\|replication protein A 70 kDa DNA-binding subunit A-like; K07466 replication factor A1 (A) | -- | -- | -- |
| 107 | BnaC01g16410D | K11855\|0\|brp:103862275\|ubiquitin carboxyl-terminal hydrolase 16; K11855 ubiquitin carboxyl-terminal hydrolase 36/42 [EC:3.4.19.12] (A) | Biological Process: nuclear-transcribed mRNA catabolic process (GO:0000956); Molecular Function: ubiquitin thiolesterase activity (GO:0004221); Molecular Function: ubiquitin-specific protease activity (GO:0004843); Molecular Function: protein binding (GO:0005515); Cellular Component: extracellular region (GO:0005576); Biological Process: protein N-linked glycosylation (GO:0006487); Biological Process: ubiquitin-dependent protein catabolic process (GO:0006511); Molecular Function: zinc ion binding (GO:0008270); Biological Process: cell proliferation (GO:0008283); Biological Process: flower development (GO:0009908); Biological Process: protein deubiquitination (GO:0016579); Biological Process: root development (GO:0048364); Biological Process: leaf development (GO:0048366); Biological Process: regulation of response to salt stress (GO:1901000); | 0.061772 | 1.043747 |
| 108 | BnaC01g17280D | K14826\|1.29241e-167\|brp:103863146\|peptidyl-prolyl cis-trans isomerase FKBP53-like; K14826 FK506-binding nuclear protein [EC:5.2.1.8] (A) | Biological Process: histone peptidyl-prolyl isomerization (GO:0000412); Biological Process: RNA methylation (GO:0001510); Molecular Function: peptidyl-prolyl cis-trans isomerase activity (GO:0003755); Molecular Function: FK506 binding (GO:0005528); Cellular Component: nucleolus (GO:0005730); Cellular Component: cytosol (GO:0005829); Biological Process: nucleosome assembly (GO:0006334); Cellular Component: membrane (GO:0016020); Biological Process: response to endoplasmic reticulum stress (GO:0034976); Molecular Function: histone binding (GO:0042393); | -- | -- |
| 109 | BnaC01g37130D | -- | Molecular Function: carboxylic ester hydrolase activity (GO:0004091); Cellular Component: extracellular region (GO:0005576); Cellular Component: vacuole (GO:0005773); Biological Process: lipid metabolic process (GO:0006629); | 0.39698 | 1.316749 |
| 110 | BnaC01g42730D | -- | Molecular Function: DNA binding (GO:0003677); Molecular Function: sequence-specific DNA binding transcription factor activity (GO:0003700); Cellular Component: nucleus (GO:0005634); Biological Process: regulation of transcription, DNA-templated (GO:0006355); Biological Process: cellular response to iron ion starvation (GO:0010106); Molecular Function: protein dimerization activity (GO:0046983); Biological Process: iron ion homeostasis (GO:0055072); | -0.33422 | 0.793212 |
| 111 | BnaC02g21870D | -- | Molecular Function: histone binding (GO:0042393); | -- | -- |
| 112 | BnaC02g34220D | -- | Cellular Component: endosome (GO:0005768); Cellular Component: vacuolar membrane (GO:0005774); Cellular Component: Golgi apparatus (GO:0005794); Cellular Component: trans-Golgi network (GO:0005802); Biological Process: transport (GO:0006810); Cellular Component: plant-type cell wall (GO:0009505); Cellular Component: plasmodesma (GO:0009506); Cellular Component: integral component of membrane (GO:0016021); | 0.065094 | 1.046153 |
| 113 | BnaC02g42090D | -- | -- | -0.11567 | 0.922954 |
| 114 | BnaC02g47270D | K04392\|2.46967e-146\|brp:103853673\|rac-like GTP-binding protein ARAC2; K04392 Ras-related C3 botulinum toxin substrate 1 (A) | Molecular Function: GTP binding (GO:0005525); Cellular Component: nucleus (GO:0005634); Cellular Component: cytoplasm (GO:0005737); Cellular Component: plasma membrane (GO:0005886); Biological Process: actin filament organization (GO:0007015); Biological Process: small GTPase mediated signal transduction (GO:0007264); Biological Process: plant-type cell wall biogenesis (GO:0009832); Biological Process: glucuronoxylan metabolic process (GO:0010413); Biological Process: xylan biosynthetic process (GO:0045492); | -- | -- |
| 115 | BnaC02g47310D | K14484\|1.43681e-121\|aly:ARALYDRAFT_496800\|auxin-induced protein IAA9; K14484 auxin-responsive protein IAA (A) | Molecular Function: sequence-specific DNA binding transcription factor activity (GO:0003700); Cellular Component: nucleus (GO:0005634); Biological Process: virus induced gene silencing (GO:0009616); Biological Process: auxin-activated signaling pathway (GO:0009734); Biological Process: vegetative phase change (GO:0010050); | 2.488624 | 5.612424 |
| 116 | BnaC03g00270D | -- | Cellular Component: plasma membrane (GO:0005886); Biological Process: vesicle-mediated transport (GO:0016192); | -0.12109 | 0.919491 |
| 117 | BnaC03g09330D | -- | Cellular Component: nucleus (GO:0005634); Biological Process: ER to Golgi vesicle-mediated transport (GO:0006888); Biological Process: sister chromatid cohesion (GO:0007062); Biological Process: reciprocal meiotic recombination (GO:0007131); Cellular Component: chloroplast (GO:0009507); Biological Process: regulation of chromosome organization (GO:0033044); Biological Process: meiotic DNA double-strand break formation (GO:0042138); Biological Process: meiotic chromosome segregation (GO:0045132); | -- | -- |
| 118 | BnaC03g10160D | -- | Cellular Component: nucleus (GO:0005634); | 0.065781 | 1.046651 |
| 119 | BnaC03g16070D | K15190\|2.10415e-168\|brp:103857196\|probable RNA methyltransferase At5g51130; K15190 7SK snRNA methylphosphate capping enzyme [EC:2.1.1.-] (A) | Biological Process: mitotic G2 phase (GO:0000085); Cellular Component: cytoplasm (GO:0005737); Molecular Function: protein methyltransferase activity (GO:0008276); Biological Process: photomorphogenesis (GO:0009640); Biological Process: cullin deneddylation (GO:0010388); Biological Process: protein ubiquitination (GO:0016567); Biological Process: histone methylation (GO:0016571); Biological Process: protein deubiquitination (GO:0016579); Biological Process: positive regulation of transcription, DNA-templated (GO:0045893); | 0.208278 | 1.155308 |
| 120 | BnaC03g29100D | -- | Cellular Component: cytoplasm (GO:0005737); Molecular Function: hydrolase activity (GO:0016787); | -- | -- |
| 121 | BnaC03g32090D | -- | Molecular Function: DNA binding (GO:0003677); Molecular Function: sequence-specific DNA binding transcription factor activity (GO:0003700); Molecular Function: protein binding (GO:0005515); Cellular Component: nucleus (GO:0005634); Biological Process: epidermal cell fate specification (GO:0009957); Biological Process: trichome patterning (GO:0048629); | -- | -- |
| 122 | BnaC03g32190D | -- | Molecular Function: binding (GO:0005488); Biological Process: regulation of transcription, DNA-templated (GO:0006355); Cellular Component: plasmodesma (GO:0009506); Biological Process: double fertilization forming a zygote and endosperm (GO:0009567); | -0.46841 | 0.722761 |
| 123 | BnaC03g32680D | -- | Cellular Component: plasma membrane (GO:0005886); Biological Process: cellulose biosynthetic process (GO:0030244); Biological Process: Golgi vesicle transport (GO:0048193); | -- | -- |
| 124 | BnaC03g40250D | K01915\|0\|brp:103859729\|glutamine synthetase cytosolic isozyme 1-3-like; K01915 glutamine synthetase [EC:6.3.1.2] (A) | Molecular Function: glutamate-ammonia ligase activity (GO:0004356); Molecular Function: copper ion binding (GO:0005507); Cellular Component: plasma membrane (GO:0005886); Biological Process: glutamine biosynthetic process (GO:0006542); Cellular Component: chloroplast (GO:0009507); Biological Process: response to sucrose (GO:0009744); Biological Process: response to glucose (GO:0009749); Biological Process: response to fructose (GO:0009750); Cellular Component: cytosolic ribosome (GO:0022626); Biological Process: nitrate assimilation (GO:0042128); Biological Process: response to cadmium ion (GO:0046686); | -0.05284 | 0.964038 |
| 125 | BnaC03g48010D | -- | Molecular Function: protein binding (GO:0005515); Molecular Function: zinc ion binding (GO:0008270); Biological Process: mRNA transcription (GO:0009299); Biological Process: response to light stimulus (GO:0009416); Cellular Component: chloroplast thylakoid membrane (GO:0009535); Cellular Component: integral component of plastid membrane (GO:0031351); | -- | -- |
| 126 | BnaC03g50700D | K14498\|0\|brp:103873775\|serine/threonine-protein kinase SRK2H; K14498 serine/threonine-protein kinase SRK2 [EC:2.7.11.1] (A) | Molecular Function: protein serine/threonine kinase activity (GO:0004674); Molecular Function: protein binding (GO:0005515); Molecular Function: ATP binding (GO:0005524); Cellular Component: nucleus (GO:0005634); Biological Process: protein phosphorylation (GO:0006468); Biological Process: response to salt stress (GO:0009651); | 0.457152 | 1.372829 |
| 127 | BnaC03g59780D | -- | Molecular Function: phosphorelay response regulator activity (GO:0000156); Molecular Function: protein histidine kinase activity (GO:0004673); Molecular Function: protein serine/threonine kinase activity (GO:0004674); Molecular Function: ATP binding (GO:0005524); Cellular Component: nucleus (GO:0005634); Cellular Component: cytoplasm (GO:0005737); Cellular Component: plasma membrane (GO:0005886); Cellular Component: plasmodesma (GO:0009506); Biological Process: embryo sac development (GO:0009553); Biological Process: cytokinin-activated signaling pathway (GO:0009736); Biological Process: phloem or xylem histogenesis (GO:0010087); Molecular Function: protein homodimerization activity (GO:0042803); Biological Process: protein autophosphorylation (GO:0046777); Biological Process: secondary growth (GO:0080117); | 0.025464 | 1.017807 |
| 128 | BnaC03g76360D | K00827\|0\|brp:103835108\|alanine--glyoxylate aminotransferase 2 homolog 1, mitochondrial; K00827 alanine-glyoxylate transaminase / (R)-3-amino-2-methylpropionate-pyruvate transaminase [EC:2.6.1.44 2.6.1.40] (A) | Cellular Component: mitochondrion (GO:0005739); Cellular Component: vacuolar membrane (GO:0005774); Cellular Component: plasma membrane (GO:0005886); Molecular Function: zinc ion binding (GO:0008270); Molecular Function: alanine-glyoxylate transaminase activity (GO:0008453); Biological Process: arginine catabolic process to glutamate (GO:0019544); Molecular Function: pyridoxal phosphate binding (GO:0030170); Biological Process: response to cadmium ion (GO:0046686); | 0.351793 | 1.276146 |
| 129 | BnaC04g14130D | K08081\|3.49063e-177\|brp:103868046\|tropinone reductase homolog At1g07440-like; K08081 Tropinone reductase 1 [EC:1.1.1.206] (A) | Molecular Function: nucleotide binding (GO:0000166); Molecular Function: oxidoreductase activity (GO:0016491); Biological Process: oxidation-reduction process (GO:0055114); | -- | -- |
| 130 | BnaC04g15860D | -- | Cellular Component: nucleus (GO:0005634); Biological Process: calcium ion transport (GO:0006816); Biological Process: cellular zinc ion homeostasis (GO:0006882); Biological Process: response to nematode (GO:0009624); | -0.02161 | 0.985134 |
| 131 | BnaC04g26180D | K00383\|0\|brp:103863219\|glutathione reductase, chloroplastic-like; K00383 glutathione reductase (NADPH) [EC:1.8.1.7] (A) | Molecular Function: glutathione-disulfide reductase activity (GO:0004362); Molecular Function: copper ion binding (GO:0005507); Molecular Function: ATP binding (GO:0005524); Cellular Component: mitochondrion (GO:0005739); Biological Process: protein targeting to mitochondrion (GO:0006626); Biological Process: glutathione metabolic process (GO:0006749); Molecular Function: electron carrier activity (GO:0009055); Biological Process: toxin catabolic process (GO:0009407); Cellular Component: chloroplast stroma (GO:0009570); Biological Process: chloroplast organization (GO:0009658); Biological Process: cell redox homeostasis (GO:0045454); Biological Process: ovule development (GO:0048481); Molecular Function: flavin adenine dinucleotide binding (GO:0050660); Molecular Function: NADP binding (GO:0050661); Biological Process: oxidation-reduction process (GO:0055114); | -- | -- |
| 132 | BnaC04g26670D | -- | Cellular Component: trans-Golgi network (GO:0005802); Cellular Component: plasma membrane (GO:0005886); Biological Process: protein targeting to membrane (GO:0006612); Biological Process: membrane fusion (GO:0006944); Biological Process: response to ethylene (GO:0009723); Biological Process: response to chitin (GO:0010200); Biological Process: regulation of plant-type hypersensitive response (GO:0010363); Biological Process: sphingolipid biosynthetic process (GO:0030148); Biological Process: negative regulation of programmed cell death (GO:0043069); Molecular Function: inositol phosphoceramide synthase activity (GO:0045140); Biological Process: defense response to fungus (GO:0050832); | -0.08245 | 0.944455 |
| 133 | BnaC04g31460D | -- | Biological Process: mitotic G2 phase (GO:0000085); Cellular Component: nucleus (GO:0005634); Biological Process: mRNA export from nucleus (GO:0006406); Biological Process: protein import into nucleus (GO:0006606); Biological Process: embryo sac egg cell differentiation (GO:0009560); Biological Process: photomorphogenesis (GO:0009640); Biological Process: regulation of flower development (GO:0009909); Biological Process: maintenance of meristem identity (GO:0010074); Biological Process: cullin deneddylation (GO:0010388); Biological Process: protein ubiquitination (GO:0016567); Biological Process: histone methylation (GO:0016571); Biological Process: protein deubiquitination (GO:0016579); Biological Process: positive regulation of transcription, DNA-templated (GO:0045893); Cellular Component: apoplast (GO:0048046); | -0.30808 | 0.807715 |
| 134 | BnaC04g31890D | -- | -- | -- | -- |
| 135 | BnaC04g32250D | -- | Cellular Component: nucleus (GO:0005634); Molecular Function: phosphoric diester hydrolase activity (GO:0008081); Biological Process: metabolic process (GO:0008152); Molecular Function: metal ion binding (GO:0046872); | 0.044559 | 1.031368 |
| 136 | BnaC04g36900D | -- | Molecular Function: protein serine/threonine kinase activity (GO:0004674); Molecular Function: ATP binding (GO:0005524); Cellular Component: plasma membrane (GO:0005886); Biological Process: protein autophosphorylation (GO:0046777); | -- | -- |
| 137 | BnaC04g38030D | -- | Cellular Component: chloroplast (GO:0009507); Biological Process: plant-type cell wall biogenesis (GO:0009832); Biological Process: cell growth (GO:0016049); Biological Process: cellulose metabolic process (GO:0030243); | 0.124979 | 1.090492 |
| 138 | BnaC04g44540D | -- | Biological Process: polysaccharide biosynthetic process (GO:0000271); Biological Process: polysaccharide catabolic process (GO:0000272); Molecular Function: protein serine/threonine kinase activity (GO:0004674); Molecular Function: ATP binding (GO:0005524); Cellular Component: plasma membrane (GO:0005886); Biological Process: starch metabolic process (GO:0005982); Biological Process: transmembrane receptor protein tyrosine kinase signaling pathway (GO:0007169); Biological Process: plant-type cell wall organization (GO:0009664); Biological Process: multidimensional cell growth (GO:0009825); Biological Process: plant-type cell wall biogenesis (GO:0009832); Biological Process: cell tip growth (GO:0009932); Biological Process: regulation of hormone levels (GO:0010817); Biological Process: glucosinolate biosynthetic process (GO:0019761); Biological Process: cellulose metabolic process (GO:0030243); Biological Process: anthocyanin accumulation in tissues in response to UV light (GO:0043481); Biological Process: protein autophosphorylation (GO:0046777); Biological Process: root hair elongation (GO:0048767); | -1.04915 | 0.483253 |
| 139 | BnaC04g47750D | -- | -- | 0.042595 | 1.029965 |
| 140 | BnaC04g52560D | -- | Molecular Function: RNA binding (GO:0003723); Molecular Function: structural constituent of ribosome (GO:0003735); Biological Process: DNA-templated transcription, elongation (GO:0006354); Biological Process: translation (GO:0006412); Cellular Component: chloroplast (GO:0009507); Cellular Component: large ribosomal subunit (GO:0015934); | -- | -- |
| 141 | BnaC04g52890D | K17839\|0\|brp:103866687\|probable polyamine oxidase 2; K17839 polyamine oxidase [EC:1.5.3.17 1.5.3.-] (A) | Biological Process: polyamine catabolic process (GO:0006598); Molecular Function: primary amine oxidase activity (GO:0008131); Biological Process: response to cold (GO:0009409); Biological Process: response to water deprivation (GO:0009414); Biological Process: response to abscisic acid (GO:0009737); Molecular Function: polyamine oxidase activity (GO:0046592); Biological Process: oxidation-reduction process (GO:0055114); | 0.168009 | 1.123507 |
| 142 | BnaC04g56270D | -- | Molecular Function: aspartic-type endopeptidase activity (GO:0004190); Biological Process: proteolysis (GO:0006508); Cellular Component: anchored component of plasma membrane (GO:0046658); | 0.023364 | 1.016327 |
| 143 | BnaC05g01650D | -- | Molecular Function: ATP binding (GO:0005524); Biological Process: biosynthetic process (GO:0009058); Cellular Component: chloroplast (GO:0009507); Molecular Function: kinase activity (GO:0016301); Biological Process: phosphorylation (GO:0016310); Molecular Function: phosphotransferase activity, alcohol group as acceptor (GO:0016773); | -- | -- |
| 144 | BnaC05g03510D | -- | -- | -0.20288 | 0.868813 |
| 145 | BnaC05g04270D | -- | Cellular Component: mitochondrion (GO:0005739); Cellular Component: vacuolar membrane (GO:0005774); Biological Process: proteolysis (GO:0006508); Molecular Function: serine-type peptidase activity (GO:0008236); | -0.22871 | 0.853397 |
| 146 | BnaC05g10580D | K09422\|0\|brp:103872133\|myb-related protein B; K09422 myb proto-oncogene protein, plant (A) | Molecular Function: chromatin binding (GO:0003682); Molecular Function: sequence-specific DNA binding transcription factor activity (GO:0003700); Cellular Component: nucleus (GO:0005634); Biological Process: regulation of transcription, DNA-templated (GO:0006355); Biological Process: guard cell differentiation (GO:0010052); Molecular Function: sequence-specific DNA binding (GO:0043565); | 0.118698 | 1.085754 |
| 147 | BnaC05g11750D | K14546\|4.93603e-124\|brp:103872336\|WD repeat-containing protein 43; K14546 U3 small nucleolar RNA-associated protein 5 (A) | Cellular Component: nucleus (GO:0005634); Biological Process: protein targeting to mitochondrion (GO:0006626); Cellular Component: plasmodesma (GO:0009506); | -0.06664 | 0.954859 |
| 148 | BnaC05g24210D | -- | Cellular Component: plasma membrane (GO:0005886); Biological Process: chloroplast organization (GO:0009658); Cellular Component: chloroplast inner membrane (GO:0009706); Biological Process: thylakoid membrane organization (GO:0010027); Biological Process: cellular cation homeostasis (GO:0030003); Biological Process: divalent metal ion transport (GO:0070838); | 0.097843 | 1.070172 |
| 149 | BnaC05g34570D | K13179\|0\|brp:103869547\|DEAD-box ATP-dependent RNA helicase 51; K13179 ATP-dependent RNA helicase DDX18/HAS1 [EC:3.6.4.13] (A) | Biological Process: RNA methylation (GO:0001510); Molecular Function: RNA binding (GO:0003723); Molecular Function: ATP binding (GO:0005524); Cellular Component: nucleolus (GO:0005730); Cellular Component: cytosol (GO:0005829); Molecular Function: ATP-dependent helicase activity (GO:0008026); Biological Process: pyrimidine ribonucleotide biosynthetic process (GO:0009220); Biological Process: regulation of flower development (GO:0009909); Biological Process: histone lysine methylation (GO:0034968); | -0.06861 | 0.953558 |
| 150 | BnaC05g34590D | K03111\|1.11711e-137\|brp:103869549\|single-stranded DNA-binding protein, mitochondrial; K03111 single-strand DNA-binding protein (A) | Molecular Function: single-stranded DNA binding (GO:0003697); Cellular Component: mitochondrion (GO:0005739); Biological Process: DNA replication (GO:0006260); | -- | -- |
| 151 | BnaC05g43980D | -- | Cellular Component: endosome (GO:0005768); Cellular Component: Golgi apparatus (GO:0005794); Cellular Component: trans-Golgi network (GO:0005802); Cellular Component: plasma membrane (GO:0005886); Biological Process: acetyl-CoA metabolic process (GO:0006084); Cellular Component: chloroplast thylakoid lumen (GO:0009543); Biological Process: plasmodesma organization (GO:0009663); Biological Process: response to abscisic acid (GO:0009737); Biological Process: response to sucrose (GO:0009744); Biological Process: response to glucose (GO:0009749); Biological Process: response to fructose (GO:0009750); Biological Process: unidimensional cell growth (GO:0009826); Biological Process: plant-type cell wall modification involved in multidimensional cell growth (GO:0009831); Biological Process: sterol biosynthetic process (GO:0016126); Biological Process: brassinosteroid biosynthetic process (GO:0016132); Molecular Function: transferase activity, transferring glycosyl groups (GO:0016757); Biological Process: cellulose biosynthetic process (GO:0030244); Biological Process: Golgi vesicle transport (GO:0048193); | 0.742771 | 1.673387 |
| 152 | BnaC05g46000D | K17604\|1.39742e-117\|brp:103870787\|uncharacterized LOC103870787; K17604 zinc finger SWIM domain-containing protein 3 (A) | -- | -- | -- |
| 153 | BnaC05g49450D | K12462\|1.32738e-145\|brp:103872030\|rho GDP-dissociation inhibitor 1-like; K12462 Rho GDP-dissociation inhibitor (A) | Molecular Function: Rho GDP-dissociation inhibitor activity (GO:0005094); Cellular Component: cytoplasm (GO:0005737); Biological Process: actin filament organization (GO:0007015); Biological Process: plant-type cell wall modification (GO:0009827); Biological Process: pollen tube growth (GO:0009860); | -0.11408 | 0.92397 |
| 154 | BnaC06g08050D | -- | -- | 3.103388 | 8.594346 |
| 155 | BnaC06g10300D | -- | Cellular Component: mitochondrion (GO:0005739); Cellular Component: vacuolar membrane (GO:0005774); Cellular Component: plastid (GO:0009536); | -0.0874 | 0.941218 |
| 156 | BnaC06g15570D | K01933\|0\|brp:103830004\|phosphoribosylformylglycinamidine cyclo-ligase, chloroplastic; K01933 phosphoribosylformylglycinamidine cyclo-ligase [EC:6.3.3.1] (A) | Molecular Function: phosphoribosylformylglycinamidine cyclo-ligase activity (GO:0004641); Molecular Function: copper ion binding (GO:0005507); Molecular Function: ATP binding (GO:0005524); Cellular Component: mitochondrion (GO:0005739); Biological Process: 'de novo' IMP biosynthetic process (GO:0006189); Biological Process: ubiquinone biosynthetic process (GO:0006744); Biological Process: pyrimidine ribonucleotide biosynthetic process (GO:0009220); Cellular Component: chloroplast stroma (GO:0009570); | 0.034147 | 1.023951 |
| 157 | BnaC06g21810D | -- | Biological Process: purine nucleobase transport (GO:0006863); Molecular Function: auxin:proton symporter activity (GO:0009672); Biological Process: auxin polar transport (GO:0009926); Biological Process: proline transport (GO:0015824); Biological Process: proton transport (GO:0015992); Cellular Component: integral component of membrane (GO:0016021); Biological Process: cysteine biosynthetic process (GO:0019344); Biological Process: transmembrane transport (GO:0055085); | -0.75217 | 0.59371 |
| 158 | BnaC06g26900D | K06890\|6.11337e-17\|brp:103868195\|BI1-like protein; K06890 (A) | Biological Process: brassinosteroid mediated signaling pathway (GO:0009742); Biological Process: unidimensional cell growth (GO:0009826); Cellular Component: integral component of membrane (GO:0016021); Molecular Function: glutamate binding (GO:0016595); | 4.842183 | 28.68417 |
| 159 | BnaC06g30530D | -- | -- | 0.172279 | 1.126837 |
| 160 | BnaC06g32380D | -- | Cellular Component: mitochondrion (GO:0005739); | -0.03764 | 0.974246 |
| 161 | BnaC06g32390D | -- | Biological Process: RNA splicing, via endonucleolytic cleavage and ligation (GO:0000394); Molecular Function: DNA binding (GO:0003677); Cellular Component: nucleus (GO:0005634); Cellular Component: mitochondrion (GO:0005739); Biological Process: DNA repair (GO:0006281); Biological Process: regulation of transcription, DNA-templated (GO:0006355); Biological Process: defense response (GO:0006952); Biological Process: methionine biosynthetic process (GO:0009086); Cellular Component: chloroplast (GO:0009507); | 0.171724 | 1.126404 |
| 162 | BnaC06g32640D | -- | Molecular Function: DNA binding (GO:0003677); Molecular Function: sequence-specific DNA binding transcription factor activity (GO:0003700); Cellular Component: nucleus (GO:0005634); Biological Process: regulation of transcription, DNA-templated (GO:0006355); Biological Process: iron ion transport (GO:0006826); Biological Process: cellular response to iron ion starvation (GO:0010106); Biological Process: response to nitrate (GO:0010167); Biological Process: vegetative to reproductive phase transition of meristem (GO:0010228); Biological Process: nitrate transport (GO:0015706); Molecular Function: protein dimerization activity (GO:0046983); Biological Process: ovule development (GO:0048481); Biological Process: lateral root development (GO:0048527); Biological Process: developmental growth (GO:0048589); Biological Process: root hair cell differentiation (GO:0048765); | -- | -- |
| 163 | BnaC06g39230D | -- | -- | -- | -- |
| 164 | BnaC06g42000D | -- | -- | 3.53245 | 11.57107 |
| 165 | BnaC07g00780D | -- | Molecular Function: DNA binding (GO:0003677); Molecular Function: sequence-specific DNA binding transcription factor activity (GO:0003700); Cellular Component: nucleus (GO:0005634); Biological Process: regulation of transcription, DNA-templated (GO:0006355); Biological Process: heme biosynthetic process (GO:0006783); Biological Process: gibberellin biosynthetic process (GO:0009686); Biological Process: gibberellic acid mediated signaling pathway (GO:0009740); Biological Process: negative gravitropism (GO:0009959); Biological Process: negative regulation of photomorphogenesis (GO:0010100); Biological Process: red light signaling pathway (GO:0010161); Biological Process: negative regulation of seed germination (GO:0010187); Molecular Function: phytochrome binding (GO:0010313); Biological Process: chlorophyll biosynthetic process (GO:0015995); Molecular Function: identical protein binding (GO:0042802); Biological Process: reproductive structure development (GO:0048608); | 0.120554 | 1.087152 |
| 166 | BnaC07g08010D | -- | Molecular Function: monooxygenase activity (GO:0004497); Molecular Function: iron ion binding (GO:0005506); Cellular Component: extracellular region (GO:0005576); Molecular Function: electron carrier activity (GO:0009055); Biological Process: cellular response to phosphate starvation (GO:0016036); Molecular Function: oxidoreductase activity, acting on paired donors, with incorporation or reduction of molecular oxygen (GO:0016705); Biological Process: galactolipid biosynthetic process (GO:0019375); Molecular Function: oxygen binding (GO:0019825); Molecular Function: heme binding (GO:0020037); Biological Process: cellular response to water deprivation (GO:0042631); Biological Process: oxidation-reduction process (GO:0055114); | -- | -- |
| 167 | BnaC07g22680D | K09487\|0\|brp:103875375\|heat shock protein 90-1-like; K09487 heat shock protein 90kDa beta (A) | Molecular Function: ATP binding (GO:0005524); Cellular Component: mitochondrion (GO:0005739); Cellular Component: vacuolar membrane (GO:0005774); Cellular Component: cytosol (GO:0005829); Biological Process: protein folding (GO:0006457); Biological Process: response to heat (GO:0009408); Biological Process: response to water deprivation (GO:0009414); Cellular Component: chloroplast stroma (GO:0009570); Biological Process: response to salt stress (GO:0009651); Biological Process: chloroplast organization (GO:0009658); Biological Process: de-etiolation (GO:0009704); Biological Process: embryo development ending in seed dormancy (GO:0009793); Cellular Component: chloroplast envelope (GO:0009941); Biological Process: response to chlorate (GO:0010157); Biological Process: protein import into chloroplast stroma (GO:0045037); Biological Process: ovule development (GO:0048481); Molecular Function: unfolded protein binding (GO:0051082); | -0.01257 | 0.991323 |
| 168 | BnaC07g25280D | K09272\|0\|brp:103875134\|FACT complex subunit SSRP1; K09272 structure-specific recognition protein 1 (A) | Biological Process: RNA splicing, via endonucleolytic cleavage and ligation (GO:0000394); Molecular Function: DNA binding (GO:0003677); Molecular Function: sequence-specific DNA binding transcription factor activity (GO:0003700); Cellular Component: nuclear euchromatin (GO:0005719); Biological Process: DNA methylation (GO:0006306); Biological Process: methylation-dependent chromatin silencing (GO:0006346); Biological Process: transcription from RNA polymerase II promoter (GO:0006366); Biological Process: cell-cell signaling (GO:0007267); Biological Process: virus induced gene silencing (GO:0009616); Biological Process: determination of bilateral symmetry (GO:0009855); Biological Process: organ morphogenesis (GO:0009887); Biological Process: meristem initiation (GO:0010014); Biological Process: vegetative phase change (GO:0010050); Biological Process: xylem and phloem pattern formation (GO:0010051); Biological Process: meristem maintenance (GO:0010073); Biological Process: vegetative to reproductive phase transition of meristem (GO:0010228); Biological Process: production of ta-siRNAs involved in RNA interference (GO:0010267); Cellular Component: FACT complex (GO:0035101); Biological Process: production of miRNAs involved in gene silencing by miRNA (GO:0035196); Biological Process: flower morphogenesis (GO:0048439); | -0.06524 | 0.955784 |
| 169 | BnaC07g25730D | -- | Cellular Component: nucleus (GO:0005634); | -0.29014 | 0.81782 |
| 170 | BnaC07g26020D | K14207\|0\|brp:103875056\|probable sodium-coupled neutral amino acid transporter 6; K14207 solute carrier family 38 (sodium-coupled neutral amino acid transporter), member 2 (A) | Biological Process: amino acid transmembrane transport (GO:0003333); Cellular Component: vacuolar membrane (GO:0005774); Cellular Component: plasma membrane (GO:0005886); Molecular Function: amino acid transmembrane transporter activity (GO:0015171); Cellular Component: integral component of membrane (GO:0016021); | 0.075043 | 1.053393 |
| 171 | BnaC07g26620D | -- | -- | 0.187203 | 1.138554 |
| 172 | BnaC07g31260D | -- | Molecular Function: Rho GTPase activator activity (GO:0005100); Cellular Component: cytoplasm (GO:0005737); Biological Process: protein targeting to vacuole (GO:0006623); Biological Process: membrane fusion (GO:0006944); Biological Process: signal transduction (GO:0007165); Biological Process: Golgi vesicle transport (GO:0048193); | 0.065174 | 1.046211 |
| 173 | BnaC07g34690D | -- | Cellular Component: extracellular region (GO:0005576); Cellular Component: mitochondrion (GO:0005739); Biological Process: proteolysis (GO:0006508); Biological Process: phosphatidylglycerol biosynthetic process (GO:0006655); Molecular Function: serine-type peptidase activity (GO:0008236); Cellular Component: chloroplast thylakoid lumen (GO:0009543); Biological Process: intracellular signal transduction (GO:0035556); | 0.083136 | 1.059318 |
| 174 | BnaC07g37770D | -- | Molecular Function: protein serine/threonine phosphatase activity (GO:0004722); Cellular Component: mitochondrion (GO:0005739); Biological Process: cellular response to phosphate starvation (GO:0016036); Biological Process: galactolipid biosynthetic process (GO:0019375); Biological Process: negative regulation of transcription, DNA-templated (GO:0045892); | -0.43193 | 0.741272 |
| 175 | BnaC07g41520D | -- | Biological Process: maltose metabolic process (GO:0000023); Molecular Function: ATP binding (GO:0005524); Biological Process: pentose-phosphate shunt (GO:0006098); Biological Process: rRNA processing (GO:0006364); Cellular Component: chloroplast stroma (GO:0009570); Biological Process: chloroplast relocation (GO:0009902); Biological Process: thylakoid membrane organization (GO:0010027); Biological Process: photosynthesis (GO:0015979); Biological Process: chlorophyll biosynthetic process (GO:0015995); Biological Process: carotenoid biosynthetic process (GO:0016117); Biological Process: iron-sulfur cluster assembly (GO:0016226); Biological Process: starch biosynthetic process (GO:0019252); Biological Process: isopentenyl diphosphate biosynthetic process, methylerythritol 4-phosphate pathway (GO:0019288); Biological Process: positive regulation of catalytic activity (GO:0043085); | 0.084144 | 1.060059 |
| 176 | BnaC07g45870D | -- | -- | -0.14776 | 0.902651 |
| 177 | BnaC07g49240D | K05539\|0\|brp:103874921\|uncharacterized LOC103874921; K05539 tRNA-dihydrouridine synthase A [EC:1.-.-.-] (A) | Biological Process: tRNA dihydrouridine synthesis (GO:0002943); Cellular Component: nucleus (GO:0005634); Biological Process: regulation of nitrogen utilization (GO:0006808); Cellular Component: chloroplast (GO:0009507); Cellular Component: phragmoplast (GO:0009524); Molecular Function: tRNA dihydrouridine synthase activity (GO:0017150); Biological Process: methylglyoxal catabolic process to D-lactate (GO:0019243); Molecular Function: flavin adenine dinucleotide binding (GO:0050660); Biological Process: oxidation-reduction process (GO:0055114); | 0.0409 | 1.028755 |
| 178 | BnaC08g05830D | -- | Molecular Function: DNA binding (GO:0003677); Molecular Function: sequence-specific DNA binding transcription factor activity (GO:0003700); Cellular Component: nucleus (GO:0005634); Biological Process: regulation of transcription, DNA-templated (GO:0006355); Biological Process: plant-type cell wall modification (GO:0009827); Biological Process: pollen tube growth (GO:0009860); Biological Process: pollen exine formation (GO:0010584); Molecular Function: protein dimerization activity (GO:0046983); | -0.08433 | 0.943223 |
| 179 | BnaC08g11060D | K03006\|0\|brp:103834927\|DNA-directed RNA polymerase II subunit 1; K03006 DNA-directed RNA polymerase II subunit RPB1 [EC:2.7.7.6] (A) | Biological Process: RNA splicing, via endonucleolytic cleavage and ligation (GO:0000394); Molecular Function: DNA binding (GO:0003677); Molecular Function: DNA-directed RNA polymerase activity (GO:0003899); Molecular Function: protein binding (GO:0005515); Cellular Component: DNA-directed RNA polymerase II, core complex (GO:0005665); Cellular Component: vacuole (GO:0005773); Biological Process: DNA methylation (GO:0006306); Biological Process: transcription from RNA polymerase II promoter (GO:0006366); Cellular Component: plasmodesma (GO:0009506); Cellular Component: chloroplast (GO:0009507); Biological Process: vegetative to reproductive phase transition of meristem (GO:0010228); Biological Process: protein desumoylation (GO:0016926); Biological Process: gene silencing by RNA (GO:0031047); Biological Process: hydrogen peroxide biosynthetic process (GO:0050665); | 0.230277 | 1.17306 |
| 180 | BnaC08g21620D | K10395\|0\|brp:103840934\|chromosome-associated kinesin KIF4A; K10395 kinesin family member 4/21/27 (A) | Molecular Function: microtubule motor activity (GO:0003777); Molecular Function: ATP binding (GO:0005524); Cellular Component: kinesin complex (GO:0005871); Cellular Component: microtubule (GO:0005874); Biological Process: microtubule-based movement (GO:0007018); Molecular Function: microtubule binding (GO:0008017); | 0.314222 | 1.243341 |
| 181 | BnaC08g24860D | -- | Cellular Component: cytosol (GO:0005829); Molecular Function: alcohol dehydrogenase (NADP+) activity (GO:0008106); Biological Process: response to cold (GO:0009409); Biological Process: response to water deprivation (GO:0009414); Cellular Component: chloroplast (GO:0009507); Biological Process: response to salt stress (GO:0009651); Molecular Function: steroid dehydrogenase activity (GO:0016229); Biological Process: response to cadmium ion (GO:0046686); Biological Process: oxidation-reduction process (GO:0055114); Molecular Function: NADP+ binding (GO:0070401); | -0.02315 | 0.984078 |
| 182 | BnaC08g32420D | -- | Cellular Component: plant-type vacuole (GO:0000325); Molecular Function: ATP binding (GO:0005524); Cellular Component: vacuolar membrane (GO:0005774); Cellular Component: Golgi apparatus (GO:0005794); Cellular Component: plasma membrane (GO:0005886); Biological Process: ATP catabolic process (GO:0006200); Biological Process: drug transmembrane transport (GO:0006855); Molecular Function: folic acid transporter activity (GO:0008517); Biological Process: response to water deprivation (GO:0009414); Cellular Component: plasmodesma (GO:0009506); Biological Process: response to wounding (GO:0009611); Biological Process: response to nematode (GO:0009624); Biological Process: systemic acquired resistance (GO:0009627); Biological Process: stomatal movement (GO:0010118); Biological Process: folic acid transport (GO:0015884); Cellular Component: integral component of membrane (GO:0016021); Biological Process: response to endoplasmic reticulum stress (GO:0034976); Molecular Function: ATPase activity, coupled to transmembrane movement of substances (GO:0042626); | 0.26377 | 1.200612 |
| 183 | BnaC08g39240D | K14312\|0\|brp:103842937\|nuclear pore complex protein Nup155-like; K14312 nuclear pore complex protein Nup155 (A) | Biological Process: RNA methylation (GO:0001510); Molecular Function: nucleocytoplasmic transporter activity (GO:0005487); Molecular Function: protein binding (GO:0005515); Cellular Component: nuclear pore (GO:0005643); Cellular Component: nucleolus (GO:0005730); Cellular Component: plasma membrane (GO:0005886); Biological Process: nucleocytoplasmic transport (GO:0006913); Cellular Component: plasmodesma (GO:0009506); Cellular Component: chloroplast (GO:0009507); Molecular Function: structural constituent of nuclear pore (GO:0017056); | -- | -- |
| 184 | BnaC08g42550D | K11092\|2.76851e-113\|brp:103836312\|U2 small nuclear ribonucleoprotein A'-like; K11092 U2 small nuclear ribonucleoprotein A' (A) | Biological Process: mRNA splicing, via spliceosome (GO:0000398); Biological Process: RNA methylation (GO:0001510); Molecular Function: nucleic acid binding (GO:0003676); Cellular Component: nucleolus (GO:0005730); Cellular Component: cytosol (GO:0005829); Biological Process: response to cold (GO:0009409); Cellular Component: chloroplast (GO:0009507); Cellular Component: Cajal body (GO:0015030); Cellular Component: ribonucleoprotein complex (GO:0030529); | -- | -- |
| 185 | BnaC08g49610D | K08244\|0\|brp:103843262\|alpha-glucan water dikinase 1, chloroplastic; K08244 alpha-glucan, water dikinase [EC:2.7.9.4] (A) | Molecular Function: protein binding (GO:0005515); Molecular Function: ATP binding (GO:0005524); Cellular Component: mitochondrion (GO:0005739); Biological Process: starch catabolic process (GO:0005983); Biological Process: circadian rhythm (GO:0007623); Cellular Component: chloroplast stroma (GO:0009570); Biological Process: response to symbiotic fungus (GO:0009610); Biological Process: cold acclimation (GO:0009631); Cellular Component: chloroplast envelope (GO:0009941); Biological Process: phosphorylation (GO:0016310); Biological Process: starch biosynthetic process (GO:0019252); Molecular Function: alpha-glucan, water dikinase activity (GO:0050521); | -8.08437 | 0.003684 |
| 186 | BnaC09g22970D | K18045\|1.66506e-126\|brp:103839853\|probable tyrosine-protein phosphatase At1g05000; K18045 tyrosine-protein phosphatase SIW14 [EC:3.1.3.48] (A) | Molecular Function: protein tyrosine phosphatase activity (GO:0004725); Cellular Component: cytoplasm (GO:0005737); Biological Process: peptidyl-tyrosine dephosphorylation (GO:0035335); Biological Process: negative regulation of MAP kinase activity (GO:0043407); Biological Process: regulation of defense response to bacterium (GO:1900424); | -0.02632 | 0.981923 |
| 187 | BnaC09g47170D | -- | -- | -- | -- |
| 188 | BnaC09g54250D | K14641\|0\|brp:103845846\|apyrase 2; K14641 apyrase [EC:3.6.1.5] (A) | Cellular Component: endosome (GO:0005768); Cellular Component: Golgi apparatus (GO:0005794); Cellular Component: trans-Golgi network (GO:0005802); Biological Process: ATP catabolic process (GO:0006200); Cellular Component: chloroplast (GO:0009507); Biological Process: pollen germination (GO:0009846); Molecular Function: ATPase activity (GO:0016887); Molecular Function: nucleoside-diphosphatase activity (GO:0017110); | 0.036036 | 1.025293 |
| 189 | BnaCnng00500D | -- | Cellular Component: nucleus (GO:0005634); | 0.160565 | 1.117725 |
| 190 | BnaCnng08310D | K00784\|0\|brp:103852919\|nuclear ribonuclease Z; K00784 ribonuclease Z [EC:3.1.26.11] (A) | Biological Process: mRNA splicing, via spliceosome (GO:0000398); Cellular Component: nucleus (GO:0005634); Biological Process: production of siRNA involved in RNA interference (GO:0030422); Biological Process: production of miRNAs involved in gene silencing by miRNA (GO:0035196); Biological Process: tRNA 3'-end processing (GO:0042780); Molecular Function: 3'-tRNA processing endoribonuclease activity (GO:0042781); Biological Process: post-translational protein modification (GO:0043687); Biological Process: positive regulation of transcription, DNA-templated (GO:0045893); | -0.42025 | 0.747296 |
| 191 | BnaCnng09710D | -- | Cellular Component: nucleus (GO:0005634); Biological Process: sister chromatid cohesion (GO:0007062); Biological Process: response to wounding (GO:0009611); Biological Process: chromatin silencing by small RNA (GO:0031048); Biological Process: meiotic chromosome segregation (GO:0045132); | -0.15907 | 0.8956 |
| 192 | BnaCnng13320D | K10606\|0\|brp:103874013\|E3 ubiquitin-protein ligase FANCL; K10606 E3 ubiquitin-protein ligase FANCL [EC:6.3.2.19] (A) | Molecular Function: ubiquitin-protein transferase activity (GO:0004842); Biological Process: DNA repair (GO:0006281); Molecular Function: zinc ion binding (GO:0008270); Biological Process: protein ubiquitination (GO:0016567); Cellular Component: Fanconi anaemia nuclear complex (GO:0043240); | -0.48225 | 0.71586 |
| 193 | BnaCnng24040D | K01595\|2.58011e-17\|eus:EUTSA_v10019997mg\|hypothetical protein; K01595 phosphoenolpyruvate carboxylase [EC:4.1.1.31] (A) | Molecular Function: protein binding (GO:0005515); Cellular Component: cytosol (GO:0005829); Biological Process: glycolytic process (GO:0006096); Biological Process: tricarboxylic acid cycle (GO:0006099); Biological Process: iron ion transport (GO:0006826); Biological Process: water transport (GO:0006833); Biological Process: hyperosmotic response (GO:0006972); Biological Process: Golgi organization (GO:0007030); Molecular Function: phosphoenolpyruvate carboxylase activity (GO:0008964); Biological Process: response to temperature stimulus (GO:0009266); Biological Process: response to salt stress (GO:0009651); Biological Process: response to sucrose (GO:0009744); Biological Process: response to glucose (GO:0009749); Biological Process: response to fructose (GO:0009750); Biological Process: cellular response to iron ion starvation (GO:0010106); Biological Process: response to nitrate (GO:0010167); Biological Process: nitrate transport (GO:0015706); Biological Process: carbon fixation (GO:0015977); Biological Process: cellular response to phosphate starvation (GO:0016036); Biological Process: response to cadmium ion (GO:0046686); Cellular Component: apoplast (GO:0048046); Biological Process: protein tetramerization (GO:0051262); | 4.770312 | 27.29021 |
| 194 | BnaCnng29650D | K07023\|2.87157e-169\|brp:103835474\|HD domain-containing protein 2 homolog; K07023 putative hydrolases of HD superfamily (A) | Molecular Function: phosphoric diester hydrolase activity (GO:0008081); Biological Process: metabolic process (GO:0008152); Cellular Component: chloroplast (GO:0009507); Molecular Function: metal ion binding (GO:0046872); | 0.134053 | 1.097372 |
| 195 | BnaCnng34210D | -- | -- | -0.04364 | 0.970203 |
| 196 | BnaCnng38200D | -- | Molecular Function: GTPase activity (GO:0003924); Molecular Function: protein binding (GO:0005515); Molecular Function: GTP binding (GO:0005525); Cellular Component: early endosome (GO:0005769); Cellular Component: Golgi apparatus (GO:0005794); Biological Process: GTP catabolic process (GO:0006184); Biological Process: protein targeting to vacuole (GO:0006623); Biological Process: membrane fusion (GO:0006944); Biological Process: small GTPase mediated signal transduction (GO:0007264); Cellular Component: chloroplast (GO:0009507); Cellular Component: cytoplasmic side of endosome membrane (GO:0010009); Biological Process: early endosome to late endosome transport (GO:0045022); | 0.436243 | 1.353076 |
| 197 | BnaCnng38630D | K00454\|0\|brp:103830995\|lipoxygenase 2, chloroplastic-like; K00454 lipoxygenase [EC:1.13.11.12] (A) | Molecular Function: iron ion binding (GO:0005506); Molecular Function: linoleate 13S-lipoxygenase activity (GO:0016165); Biological Process: oxylipin biosynthetic process (GO:0031408); Biological Process: oxidation-reduction process (GO:0055114); | -- | -- |
| 198 | BnaCnng40140D | K10575\|1.00451e-112\|brp:103830011\|ubiquitin-conjugating enzyme E2 14; K10575 ubiquitin-conjugating enzyme E2 G1 [EC:6.3.2.19] (A) | Molecular Function: ubiquitin-protein transferase activity (GO:0004842); Cellular Component: nucleus (GO:0005634); Biological Process: ubiquitin-dependent protein catabolic process (GO:0006511); Biological Process: protein ubiquitination (GO:0016567); | -0.17181 | 0.88773 |
| 199 | BnaCnng54620D | K08235\|0\|brp:103865563\|probable xyloglucan endotransglucosylase/hydrolase protein 32; K08235 xyloglucan:xyloglucosyl transferase [EC:2.4.1.207] (A) | Molecular Function: hydrolase activity, hydrolyzing O-glycosyl compounds (GO:0004553); Cellular Component: cell wall (GO:0005618); Biological Process: cellular glucan metabolic process (GO:0006073); Molecular Function: xyloglucan:xyloglucosyl transferase activity (GO:0016762); Biological Process: cell wall macromolecule catabolic process (GO:0016998); Biological Process: cell wall biogenesis (GO:0042546); Cellular Component: apoplast (GO:0048046); | 0.29623 | 1.227931 |
| 200 | BnaCnng56430D | -- | Cellular Component: endosome (GO:0005768); Cellular Component: Golgi apparatus (GO:0005794); Cellular Component: trans-Golgi network (GO:0005802); Cellular Component: cytosol (GO:0005829); Cellular Component: plasma membrane (GO:0005886); Biological Process: protein transport (GO:0015031); Cellular Component: integral component of membrane (GO:0016021); Molecular Function: transmembrane transporter activity (GO:0022857); Biological Process: transmembrane transport (GO:0055085); | -0.05775 | 0.960759 |
| 201 | BnaCnng59870D | -- | Biological Process: response to hypoxia (GO:0001666); Molecular Function: ATP binding (GO:0005524); Biological Process: systemic acquired resistance, salicylic acid mediated signaling pathway (GO:0009862); Biological Process: regulation of hydrogen peroxide metabolic process (GO:0010310); Molecular Function: nucleoside-triphosphatase activity (GO:0017111); Biological Process: regulation of defense response (GO:0031347); Molecular Function: ADP binding (GO:0043531); | 0.009961 | 1.006928 |
| 202 | BnaCnng61530D | K17686\|0\|brp:103839159\|copper-transporting ATPase RAN1-like; K17686 Cu+-exporting ATPase [EC:3.6.3.54] (A) | Biological Process: response to superoxide (GO:0000303); Molecular Function: GTPase activity (GO:0003924); Molecular Function: copper ion binding (GO:0005507); Molecular Function: protein binding (GO:0005515); Molecular Function: ATP binding (GO:0005524); Molecular Function: GTP binding (GO:0005525); Cellular Component: cell wall (GO:0005618); Cellular Component: nucleus (GO:0005634); Cellular Component: endosome (GO:0005768); Cellular Component: Golgi apparatus (GO:0005794); Cellular Component: trans-Golgi network (GO:0005802); Cellular Component: cytosol (GO:0005829); Cellular Component: plasma membrane (GO:0005886); Biological Process: protein N-linked glycosylation (GO:0006487); Biological Process: protein import into nucleus (GO:0006606); Biological Process: ATP biosynthetic process (GO:0006754); Biological Process: cell death (GO:0008219); Biological Process: response to salt stress (GO:0009651); Biological Process: salicylic acid mediated signaling pathway (GO:0009863); Biological Process: ethylene-activated signaling pathway (GO:0009873); Biological Process: regulation of stomatal movement (GO:0010119); Cellular Component: integral component of membrane (GO:0016021); Biological Process: copper ion transmembrane transport (GO:0035434); Molecular Function: copper-transporting ATPase activity (GO:0043682); Biological Process: response to cadmium ion (GO:0046686); Cellular Component: apoplast (GO:0048046); | -0.14646 | 0.903463 |
| 203 | BnaCnng68400D | K11644\|4.32012e-65\|eus:EUTSA_v10012461mg\|hypothetical protein; K11644 paired amphipathic helix protein Sin3a (A) | Cellular Component: nucleus (GO:0005634); Cellular Component: cytosol (GO:0005829); Biological Process: regulation of transcription, DNA-templated (GO:0006355); Biological Process: vernalization response (GO:0010048); Biological Process: anthocyanin accumulation in tissues in response to UV light (GO:0043481); Biological Process: carpel development (GO:0048440); | -- | -- |
| 204 | BnaUnng01520D | -- | Cellular Component: nucleus (GO:0005634); Biological Process: protein import into nucleus (GO:0006606); Biological Process: pyrimidine ribonucleotide biosynthetic process (GO:0009220); | -0.1117 | 0.925496 |
| 205 | BnaUnng05070D | K07466\|2.08302e-23\|brp:103874121\|replication protein A 70 kDa DNA-binding subunit B-like; K07466 replication factor A1 (A) | -- | -- | -- |
